# Comparative Mitogenomic Analyses of Darkling Beetles (Coleoptera: Tenebrionidae) Provide Evolutionary Insights into tRNA-like Sequences

**DOI:** 10.3390/genes14091738

**Published:** 2023-08-30

**Authors:** Su-Hao Wang, Shi-Yun Hu, Min Li, Min Liu, Hao Sun, Jia-Rui Zhao, Wen-Ting Chen, Ming-Long Yuan

**Affiliations:** 1 State Key Laboratory of Herbage Improvement and Grassland Agro-Ecosystems, Lanzhou University, Lanzhou 730020, China; wangsh21@lzu.edu.cn (S.-H.W.); hushy20@lzu.edu.cn (S.-Y.H.); limin2015@lzu.edu.cn (M.L.); mliu19@lzu.edu.cn (M.L.); sunh2021@lzu.edu.cn (H.S.); zhaojr21@lzu.edu.cn (J.-R.Z.); chenwt18@lzu.edu.cn (W.-T.C.); 2 Key Laboratory of Grassland Livestock Industry Innovation, Ministry of Agriculture and Rural Affairs, Lanzhou University, Lanzhou 730020, China; 3 College of Pastoral Agricultural Science and Technology, Lanzhou University, Lanzhou 730020, China; 4 National Demonstration Center for Experimental Grassland Science Education, Lanzhou University, Lanzhou 730020, China

**Keywords:** Tenebrionidae, mitochondrial genome, tRNA-like sequence, control region, phylogenetic analysis

## Abstract

Tenebrionidae is widely recognized owing to its species diversity and economic importance. Here, we determined the mitochondrial genomes (mitogenomes) of three Tenebrionidae species (*Melanesthes exilidentata*, *Anatolica potanini*, and *Myladina unguiculina*) and performed a comparative mitogenomic analysis to characterize the evolutionary characteristics of the family. The tenebrionid mitogenomes were highly conserved with respect to genome size, gene arrangement, base composition, and codon usage. All protein-coding genes evolved under purifying selection. The largest non-coding region (i.e., control region) showed several unusual features, including several conserved repetitive fragments (e.g., A+T-rich regions, G+C-rich regions, Poly-T tracts, TATA repeat units, and longer repetitive fragments) and tRNA-like structures. These tRNA-like structures can bind to the appropriate anticodon to form a cloverleaf structure, although base-pairing is not complete. We summarized the quantity, types, and conservation of tRNA-like sequences and performed functional and evolutionary analyses of tRNA-like sequences with various anticodons. Phylogenetic analyses based on three mitogenomic datasets and two tree inference methods largely supported the monophyly of each of the three subfamilies (Stenochiinae, Pimeliinae, and Lagriinae), whereas both Tenebrioninae and Diaperinae were consistently recovered as polyphyletic. We obtained a tenebrionid mitogenomic phylogeny: (Lagriinae, (Pimeliinae, ((Tenebrioninae + Diaperinae), Stenochiinae))). Our results provide insights into the evolution and function of tRNA-like sequences in tenebrionid mitogenomes and contribute to our general understanding of the evolution of Tenebrionidae.

## 1. Introduction

Tenebrionidae is a large family of Coleoptera, with more than 20,000 extant species in eight to ten subfamilies [1,2,3]. Species of Tenebrionidae are widely distributed worldwide and are extremely well-adapted to their environments [4]. Complex feeding strategies and habitats lead to diverse morphological features and economic impacts of species in this family. Many tenebrionid species are agricultural pests; for example, *Tribolium* spp. are destructive cosmopolitan pests of stored flour, corn, peanuts, and other dried agricultural products [5]; however, its larvae are rich in protein and can be used as feeds for pets, such as reptiles and birds [6]. Some tenebrionid species are also valuable for ecological improvement. For example, *Tenebrio molitor* Linnaeus and *Uloma* spp. can biodegrade polystyrene (PS) by acting synergistically with intestinal microbiota [7,8].

During the past few decades, the phylogenetic relationships within Tenebrionidae have been investigated extensively based on both morphological [2,3,9,10] and molecular data [1,4,11,12,13,14]. However, the structure and relationships among many subfamilies of Tenebrionidae are not well-resolved [14]. Hunt et al. (2007) reconstructed a large-scale beetle phylogeny; however, Lagriinae and Pimeliinae did not group with the remaining subfamilies in Tenebrionidae [15]. Kergoat et al. (2014) utilized maximum likelihood to construct the Tenebrionidae phylogeny and found that each of the three subfamilies (Lagriinae, Alleculinae, and Stenochiinae) were monophyletic, whereas Pimeliinae was paraphyletic [16]. A recent phylogenetic analysis based on 37 species showed that Tenebrioninae and Diaperinae were polyphyletic, while the monophyly of four subfamilies (Lagriinae, Alleculinae, Stenochiinae, and Pimeliinae) could not be resolved using RAxML and MrBayes [17]. Further studies of phylogenetic relationships within Tenebrionidae are needed.

The tRNA-like structure was first identified in the RNA genome of *turnip yellow mosaic virus* [18]. Most tRNA-like structures have been found in plant viruses and their functions have been studied in depth [19,20,21]. However, some have been found in animals, such as a tRNA-like structure in human immunodeficiency virus type 1 (HIV-1) that can serve as a primer for reverse transcription of the HIV-1 genome [22]. In addition to viruses, tRNA-like structures have been detected in RNAs from many different species [23,24,25]. However, few tRNA-like sequences have been reported in insect mitochondrial genomes (mitogenomes) [26,27,28], and their functional and evolutionary significance are not well studied. 

The insect mitogenome is a compact double-stranded circular molecule of 15–18 kb that encodes 37 genes, including 13 protein-coding genes (PCGs), two ribosomal RNA genes (rRNAs), and 22 transfer RNA genes (tRNAs) [29,30], as well as a large non-coding region (i.e., the control region, also termed the A+T-rich region in insects), which contains the origin of replication [31]. Owing to its simple genetic structure, matrilineal inheritance, and high copy number, insect mitogenomes are commonly used for phylogenetics, molecular evolutionary, and conservation genetics research [11,29,32]. Mitogenome sequences of 53 tenebrionid species have been deposited in GenBank (as of February 2023). However, several mitogenomes were not validated and lacked some genes or A+T-rich regions. Additional tenebrionid mitogenomes will be helpful for phylogenetic and comparative mitogenomic analyses of Tenebrionidae, especially for clarifying the evolutionary features of mitochondrial non-coding regions.

In this study, we sequenced and annotated the complete mitogenomes of three tenebrionid species, i.e., *Melanesthes exilidentata* Ren, *Anatolica potanini* Reitter, and *Myladina unguiculina* Reitter. We performed a comparative mitogenomic analysis, including analyses of the nucleotide composition, codon usage, tRNA secondary structure, and evolutionary rates of 13 PCGs. We also evaluated phylogenetic relationships among six subfamilies within Tenebrionidae based on three mitogenomic datasets and two tree inference methods. In addition, we comprehensively analyzed the structural features of A+T-rich regions, with a focus on the evolutionary characteristics of tRNA-like structures. The results of this study provide new insights into the phylogeny and evolution of Tenebrionidae from a mitogenomic perspective.

## 2. Materials and Methods

### 2.1. Sampling and DNA Extraction

Adult specimens of *M. exilidentata*, *A. potanini*, and *M. unguiculina* were collected from Lingwu City, Ningxia Hui Autonomous Region. Detailed sampling information is shown in Table 1. Samples and voucher specimens have been deposited in the College of Pastoral Agricultural Science and Technology, Lanzhou University, Lanzhou, China. All specimens were initially preserved in 100% ethanol at the sampling site and then transferred to −80 °C until used for DNA extraction. Total genomic DNA was extracted from a single specimen using a DNeasy Tissue Kit (Qiagen, Hilden, Germany) according to the manufacturer’s protocols. The brief protocols were as follows: approximately 25 mg tissue were placed in a 1.5 mL microcentrifuge tube and ground with liquid nitrogen; 180 µL buffer ATL and 20 µL Proteinase K were added to the tube, and then incubated at 56 °C for 2 h; 200 µL buffer AL was added to the tube and mixed thoroughly by vortexing; the mixture was pipetted into the DNeasy Mini spin column and placed in a 2 mL collection tube and centrifuged at 8000× *g* for 1 min; the DNeasy Mini spin column was placed in a new 2 mL collection tube, 500 µL buffer AW1 was added, and centrifuged for 1 min at 8000× *g*; the DNeasy Mini spin column was placed in a new 2 mL collection tube, 500 µL buffer AW2 was added and then centrifuged for 3 min at 20,000× *g* to dry the DNeasy membrane; the DNeasy Mini spin column was placed in a clean 2 mL microcentrifuge tube, 200 µL buffer AE was pipetted directly onto the DNeasy membrane, then incubated at room temperature for 2 min, and finally centrifuged for 1 min at 8000× *g*. The quality of the extracted DNA was evaluated by 1.2% agarose gel electrophoresis and spectrophotometry with a NanoDrop ND-1000 (Thermo Scientific, Waltham, MA, USA).

### 2.2. Mitogenome Sequencing, Assembly, and Annotation

The mitogenomes of the three species were sequenced using the Illumina NovaSeq 6000 (2 × 150 bp) platform by the Wuhan Benagen Tech Solutions Company Limited (Wuhan, China). Low-quality reads were removed using SOAPnuke (version: 2.1.0) [33] and high-quality reads were assembled using SPAdes (version: 3.13.0) [34]. The three assembled mitogenomes were annotated with the MITOS web server (http://mitos2.bioinf.uni-leipzig.de; accessed on 12 March 2022) [35] to identify 13 PCGs, 22 tRNAs, and two rRNAs using the mitogenomes from Tenebrionidae in GenBank as references. To avoid overlap between genes, all genes were manually verified and proofread after annotation. Then, the online Tandem Repeats Finder web tool (version: 4.09) (https://tandem.bu.edu/trf/trf.html; accessed on 20 May 2022) was used to predict tandem repeats in the control regions. The three newly obtained mitogenome sequences have been deposited in NCBI (GenBank accession numbers OQ534869, OQ511732, and OQ536315).

### 2.3. Comparative Mitogenomic Analysis of Tenebrionidae

Thirty-three Tenebrionidae mitogenomes were used to compare the full-length, size-differentiated variation and to calculate the genome-level base composition. MEGA 11 [36] was used to calculate the base composition of each PCG, including the overall composition and values at the first, second, and third loci. The number of codons and relative synonymous codon usage (RSCU) of the 13 PCGs of each species were calculated using MEGA 11, and results for each species were output individually and used to analyze differences in the most-used codons with respect to the A+T content. Asymmetry was evaluated using the following formula: AT-skew = (A − T)/(A + T), GC-skew = (G − C)/(G + C) [37]. The effective codon number (ENC) and codon bias index (CBI) were determined for each Tenebrionidae mitogenome using DnaSP [38]. To compare codon usage bias among these 34 species, the correlations between ENC, CBI, G+C content of all codons, and G+C content at the third codon position (GC3) were evaluated. Based on the ENC and GC3 for each species, a parabolic curve was fitted to ENC, providing a basis for evaluating whether codon usage was subject to natural selection [39]. We analyzed the control (AT-rich) and non-coding regions of the 33 tenebrionid species, focusing on their specific features (tRNA-like structures) and potential evolutionary significance. We also calculated the rates of synonymous substitutions (*K_s_*) and non-synonymous substitutions (*K_a_*) for each PCG using MEGA 11 [36].

### 2.4. Phylogenetic Analysis

Mitogenome sequences of 33 tenebrionid species and one outgroup (Meloidae) were used for a phylogenetic analysis. Details of the species included in this study are given in Table S1. All 13 PCGs were individually aligned by ClustalW (Codons) and each of the two rRNAs (*rrnL* and *rrnS*) were aligned by ClustalW, implemented in MEGA 11 [36]. Three mitogenome datasets were constructed for the phylogenetic analysis: (1) the P123 dataset, with nucleotide sequences for all codon positions of 13 PCGs, including 11,172 nucleotides; (2) the P123AA dataset, with inferred amino acid sequences of 13 PCGs, including 3724 amino acids; and (3) the P123RNA dataset, with P123 and nucleotide sequences of two rRNAs, comprising 13379 nucleotides. DAMBE 5.3.74 [40] was used for substitution saturation tests, revealing that none of the 13 PCGs had significant substitution saturation (Table S2). The IQ-TREE web server (http://iqtree.cibiv.univie.ac.at/; accessed on 2 July 2022) was used to determine the best partitioning schemes and corresponding nucleotide substitution model for each of the three datasets, and the results (Table S3) were used for the phylogenetic analysis.

We used RAxML-HPC2 [41] to construct maximum likelihood (ML) phylogenetic trees of Tenebrionidae, with the GTRGAMMAI model and 1000 bootstrap replicates (BS). Additionally, MrBayes 3.2.7 [42] was used for Bayesian inference (BI), employing four independent Markov chains, including three heated chains and one cold chain, and running 1 × 10^8^ generations concurrently. When the estimated sample size was greater than 100 and the potential size reduction factor was close to 1.0, chain convergence was achieved [42].

## 3. Results

### 3.1. General Characteristics of Tenebrionidae Mitogenomes

**Figure 1:**
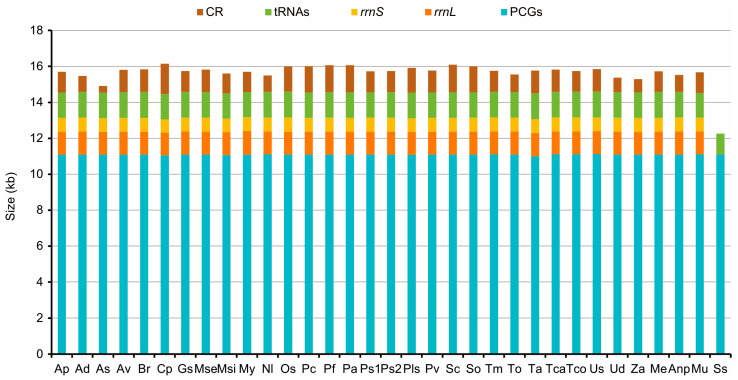
Sizes of PCGs, tRNAs, *rrnL*, *rrnS*, and CR in Tenebrioidae mitogenomes. Species are abbreviated as follows: *Adelium* sp, Ap; *Alphitobius diaperinus*, Ad; *Amarygmini* sp, As; *Asbolus verrucosus*, Av; *Blaps rhynchoptera*, Br; *Cerogria popularis*, Cp; *Gonocephalum* sp, Gs; *Machla setosa*, Mse; *Morphostenophanes sinicus*, Msi; *Morphostenophanes yunnanus*, My; *Nalassus laevioctostriatus*, Nl; *Opatrum sabulosum*, Os; *Pelecyphorus contortus*, Pc; *Pelecyphorus foveolatus*, Pf; *Philolithus aegrotus*, Pa; *Philolithus* sp1, Ps1; *Philolithus* sp2, Ps2; *Platydema* sp, Pls; *Promethis valgipes*, Pv; *Stenomorpha consobrina*, Sc; *Stenomorpha obovata*, So; *T. molitor*, Tm; *Tenebrio obscurus*, To; *Tribolium audax*, Ta; *Tribolium castaneum*, Tca; *Tribolium confusum*, Tco; *Uloma* sp, Us; *Ulomoides dermestoides*, Ud; *Zophobas atratus*, Za; *M. exilidentata*, Me; *A. potanini*, Anp; *M. unguiculina*, Mu; *Strongylium suspicax*, Ss.

**Figure 2:**
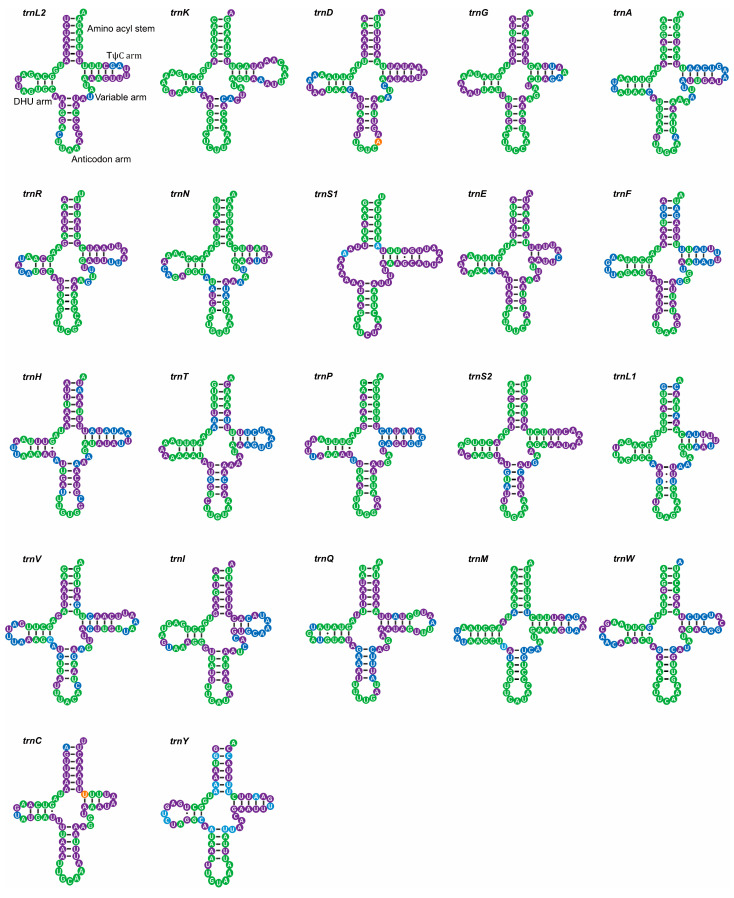
Putative secondary structures of 22 tRNA genes identified in the mitogenome of *M. unguiculina*. All tRNAs are shown in the order of occurrence in the mitogenome starting from *trnL2*. Bars indicate Waston-Crick base pairings, and dots between G and U pairs mark canonical base pairings in tRNA. Different colors represent different levels of base conservation. Green indicates conservation among all Tenebrionidae, orange indicates conservation among subfamilies, purple indicates conservation among the three newly sequenced species, and blue indicates a lack of conservation among the three newly sequenced species.

The mitogenomes were 15542 bp in *M. unguiculina*, 15804 bp in *M. exilidentata*, and 15,809 bp in *A. potanini* (Figure 1), and all were typical closed-loop DNA molecules containing 37 genes (13 PCGs, 22 tRNAs, and 2 rRNAs) and containing a large non-coding region. The mitogenomes of all three species had a typical invertebrate mitochondrial gene arrangement. Fourteen genes (four PCGs, eight tRNAs, and two rRNAs) were transcribed from the minority strand (N strand), leaving 23 genes (nine PCGs and 14 tRNAs) transcribed from the majority strand (J strand). In all three Tenebrionidae mitogenomes, there was substantial gene overlap at *atp6*/*atp8* (7 bp) and *nad4*/*nad4L* (7 bp or 8 bp) (Table S4), and many intergeneric regions (generally ≤3 bp) were widely distributed. A large intergeneric space (16 bp to 20 bp) was found between *trnS2* (UCN) and *nad1* in all three Tenebrionidae mitogenomes. In addition, *M. exilidentata* and *A. potanini* had a large intergeneric space (47 bp) between *trnC* and *trnW*, while *M. unguiculina* had a 19 bp intergeneric space between *nad5* and *trnH* (Table S4). Except *trnS1*, which lacked the dihydrouridine (DHU) arm, the remaining 21 tRNAs possessed a canonical cloverleaf secondary structure, consisting of four arms. The anticodon arms and DHU stems of all tRNAs were highly conserved, with most substitutions and differences present in the DHU loop, the pseudouridine (TψC) arm, and the variable loop (Figure 2).

**Figure 3:**
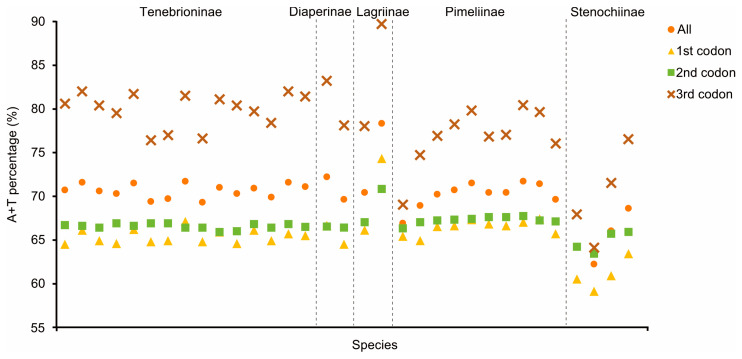
A+T% of the mitochondrial protein-encoding genes in the three groups within the 33 Tenebrionidae species. The dashed line separates the different subfamilies.

Apart from *nad1* in three Tenebrionidae mitogenomes, all PCGs started with typical ATN codons, while all PCGs ended with complete termination codons (TAA/TAG) or truncated codons (TA or T) (Table S4). Among the 13 PCGs in the three mitogenomes, *cox1* had the lowest A+T content (65.43–65.70%) and *atp8* had the highest (79.49–81.41%). A component analysis of the 13 PCGs from 33 species showed that the third codon position had the highest A+T content, while the first and second codon positions had lower A+T contents (Figure 3). *K_a_*/*K_s_* values of 13 PCGs in the 33 tenebrionid species were less than 1.0 (Figure S1); *nad6* (0.37) and *atp8* (0.36) showed the highest *K_a_* values, while *cox1* (0.05) had the lowest. There was a negative correlation between the *K_a_*/*K_s_* values and the G+C content of 13 PCGs (*R*^2^ = 0.86, *p* < 0.01; Figure S2).

### 3.2. Nucleotide Composition and Codon Usage

**Figure 4:**
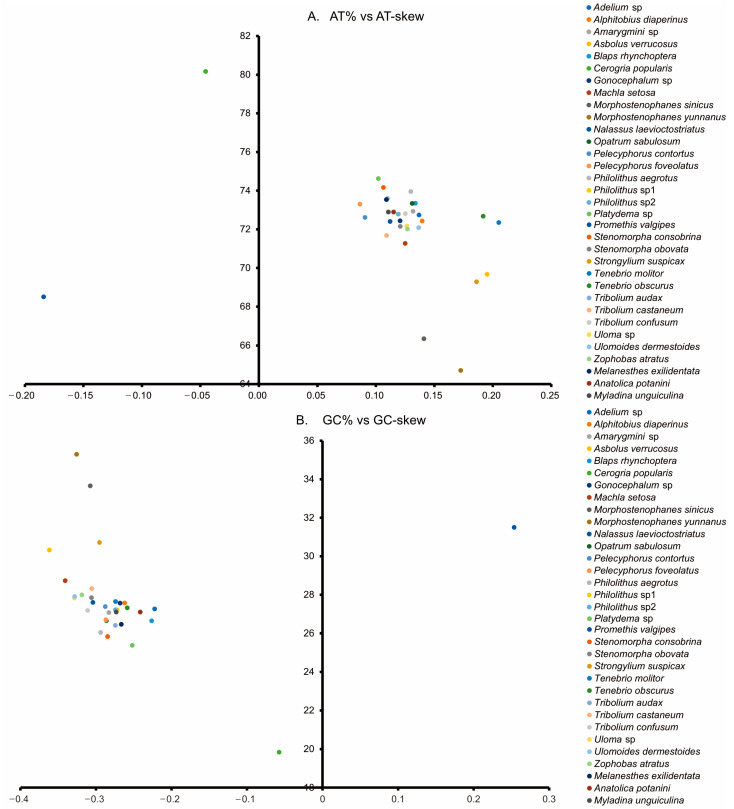
AT% vs. AT-skew (**A**) and GC% vs. GC-skew (**B**) in the 33 mitogenomes of the Tenebrionidae and one mitogenome of the outgroup. Measured in bp percentage (Y-axis) and level of nucleotide skew (X-axis). Values are calculated on J-strands for full-length mitochondrial genomes.

The nucleotide compositions of the three new Tenebrionidae species were biased toward A and T, with A+T contents ranging from 71.0% to 71.5% (Figure 3). AT-skew ranged from 0.108 in *M. exilidentata* to 0.115 in *A. potanini*, while GC-skew ranged from −0.273 in *M. unguiculina* to −0.241 in *A. potanini* (Figure 4). The high A+T content and nucleotide bias in the mitogenomes were also reflected in the codon usage of PCGs. An RSCU analysis revealed that four AT-rich codons, i.e., AUU(I), UUU(F), UUA(L), and AUA(M), were used most frequently (Table S5). Except for the *M. unguiculina* mitogenome, which used 61 codons, both *M. exilidentata* and *A. potanini* used all 62 invertebrate codons.

**Figure 5:**
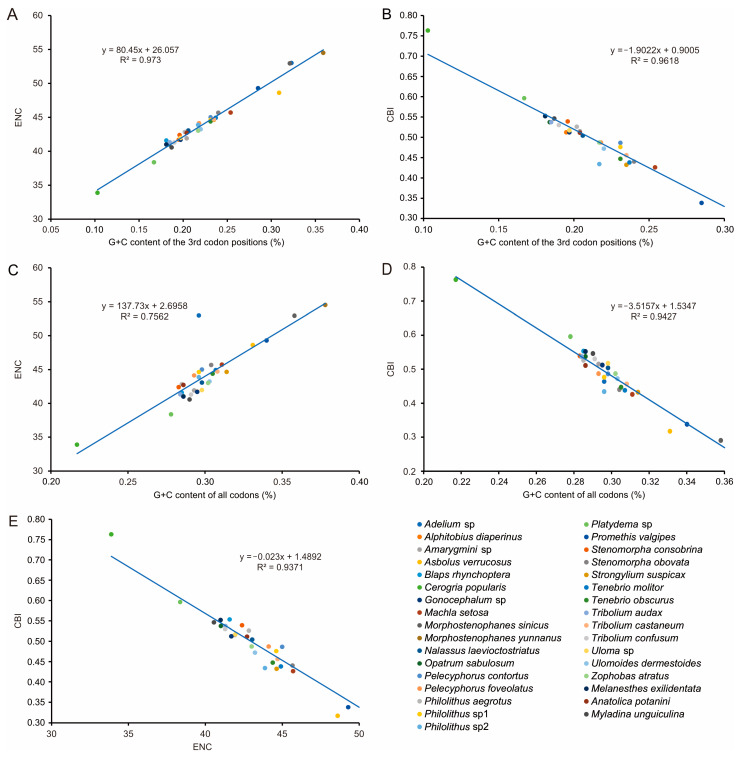
Evaluation of codon bias in the mitochondrial genomes of 33 Tenebrionidae species. (**A**) the correlation between ENC (effective number of codons) and the G + C content of the 3rd codon positions. (**B**) the correlation between CBI (codon bias index) and the 3rd codon positions. (**C**) the correlation between ENC and the G + C content of all codons. (**D**) the correlation between CBI and the G + C content of all codons. (**E**) the correlation between ENC and CBI.

**Figure 6:**
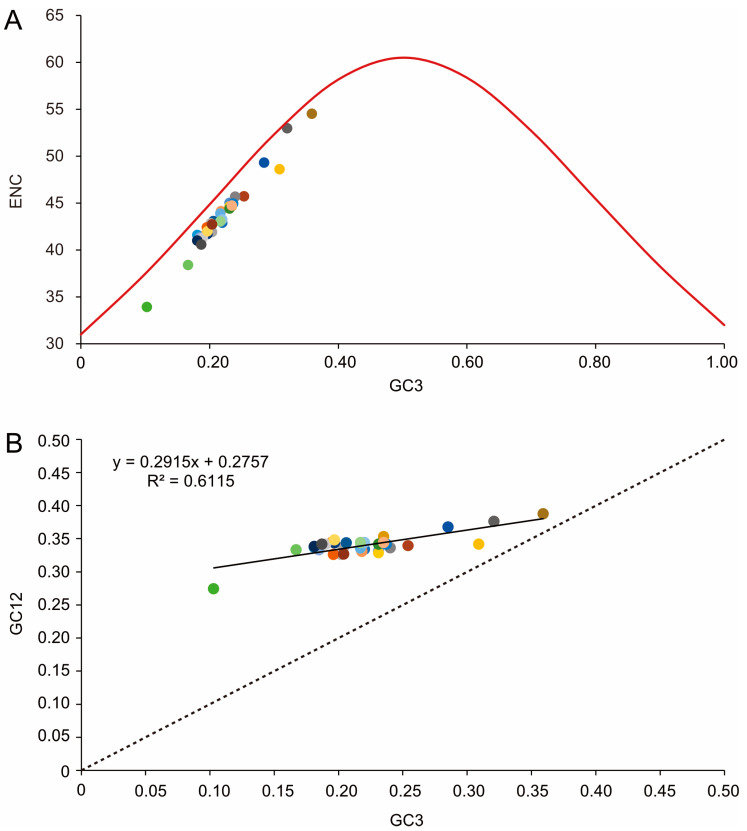
(**A**) Correlations between the effective number of codons (ENC) and G+C content of the third codon positions (GC3) for the 33 Tenebrionidae species. The solid line represents the relationship between the ENC* (2 + GC3 + (29/[(GC3)^^2^ + (1 − GC3)^^2^]) and the GC3. (**B**) The solid line represents the relationship between the GC3 and G+C content of the first and second positions (GC12), whereas the dotted line indicates y = x. Each color dot represents a tenebrionid species and colors match those in Figure 4.

To further investigate codon usage bias, the ENC and G+C content were analyzed for 13 PCGs of all 33 tenebrionid species. The mean ENC value for all species was 44.03, with a range of 33.89–54.51, indicating a strong codon bias. Positive correlations were found between the ENC of all codons and GC content of the third codon positions (GC3) (*R*^2^ = 0.97, *p* < 0.01) (Figure 5A) and G+C content (*R*^2^ = 0.76, *p* < 0.01) (Figure 5C. In addition, negative correlations were found between CBI and GC3 (*R*^2^ = 0.96, *p* < 0.01) (Figure 5B), G+C content at all positions (GC) (*R*^2^ = 0.94, *p* < 0.01) (Figure 5D), and ENC (*R*^2^ = 0.94, *p* < 0.01) (Figure 5E). The ENC values for all Tenebrionidae species were below the ENC curve (Figure 6A) and no significant correlation (*R*^2^ = 0.61, *p* > 0.05) was found between GC12 and GC3 (Figure 6B).

### 3.3. Non-Coding Region

**Figure 7:**
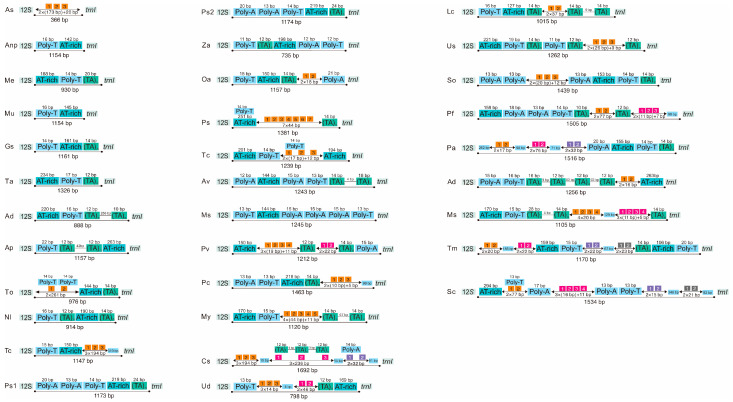
Organization of the control regions in the Tenebrionidae mitogenomes. Location and copy number of tandem repeats are shown in orange and three colors (pink, purple, and grey) with Arabic numbers inside. Sky blue boxes represent interval sequences (positive numbers) or overlap (negative numbers) between two elements. Green boxes represent TA tandem repeat sequences. See Figure 1 for the full names of species.

The mitochondrial control region (A+T-rich region), the largest non-coding region, was located between *rrnS* and *trnI* (Figure 7), with a length of 930 bp in *M. unguiculina* and 1154 bp in both *M. exilidentata* and *A. potanini*. All 33 sequenced tenebrionid mitogenomes included a control region, with a maximum length difference of 1326 bp (Figure 7). The A+T content of control regions ranged from 80.59% in *B. rhynchoptera* Fairmaire to 94.34% in *T. molitor*. Several features were found in the control region of the 33 tenebrionid species, including A+T-rich regions (83.51–88.97%), G+C-rich regions, Poly-T stretches, TATA repeat units, as well as longer repeat fragments (1–4 repeats).

We also found tRNA-like structures in the tenebrionid control regions. One, two, and three tRNA-like structures were detected in *M. exilidentata*, *M. unguiculina*, and *A. potanini*. Among the 33 tenebrionid species used for phylogenetic analyses, 22 species had at least one tRNA-like structure (Table S6), and the number of tRNA-like structures ranged from 1 to 9 (Table S7). The two most abundant tRNA-like structures were *trnL2*-like (nine) and *trnW*-like (seven), followed by the *trnV*-like and *trnE*-like structures, with five and four occurrences, respectively. The remaining tRNA-like sequences were only discovered in one to two cases (Table S7). These two tRNA-like structures (*trnL2*-like and *trnW*-like) were commonly found in tenebrionid species belonging to five subfamilies (Table S7).

**Figure 8:**
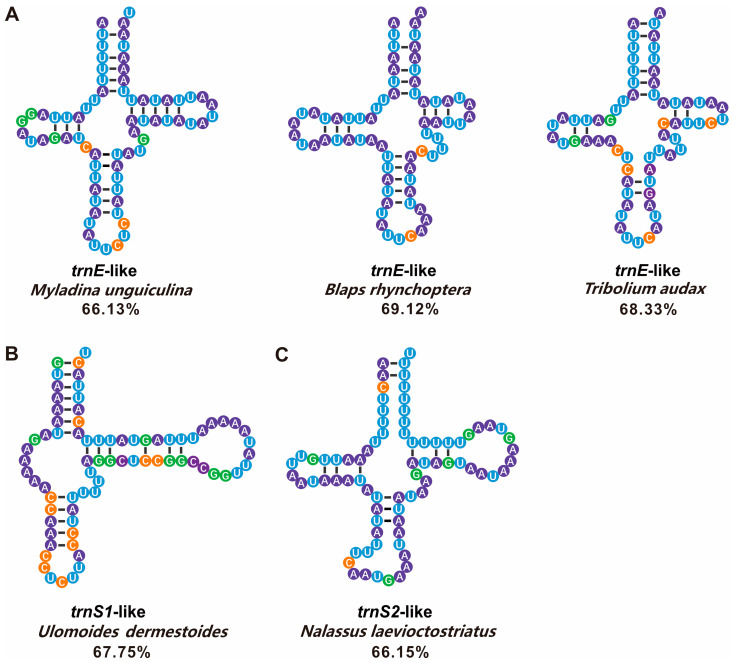
Five tRNA-like secondary structures with a match of 65% or more were selected from the A+T-rich regions of 33 Tenebrionidae species, which were involved in the construction of the phylogenetic tree. (**A**) Three *trnE*-like sequences. (**B**) one *trnS1*-like sequence. (**C**) one *trnS2*-like sequence. The tRNAs are labeled with the abbreviations of their corresponding amino acids. A dash (-) indicates Watson-Crick base-pairing. Blue, purple, green, and orange represent U, A, G, and C bases, respectively. Percentages represent the consensus sequences of tRNA-like sequences compared to each corresponding conventional tRNA.

Only three Pimeliinae species possessed both *trnL2*-like and *trnW*-like. These tRNA-like structures possessed the appropriate anticodons but generally lacked conserved base pairing arms, forming a cloverleaf secondary structure. We predicted several tRNA-like structures matches of greater than 65% with the corresponding conventional tRNAs (Figure 8). These had a relatively stable cloverleaf structure and more complete base pairing. Furthermore, tRNA-like sequences with less than a 65% match were aligned with the equivalent conventional tRNAs by tagging the same anticodon (Figure S3).

In addition to the control region, we identified several spacer regions of different lengths, including a highly conserved intergenic spacer sequence between *trnS2* and *nad1*, which was found in all 33 Tenebrionidae species and was 15–19 bp in size, with three highly conserved fragments (motif I, motif II, and motif III) (Figure S4).

### 3.4. Mitochondrial Phylogeny of Tenebrionidae

**Figure 9:**
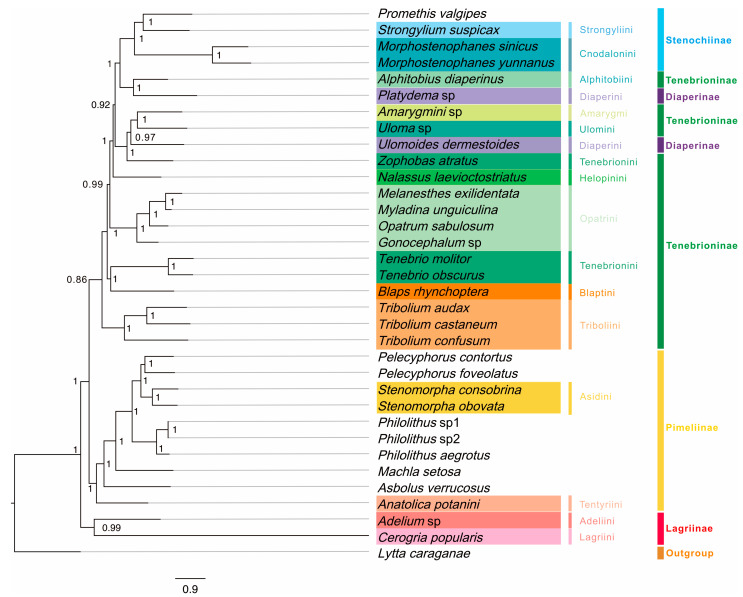
Mitogenome-based phylogenetic relationships among 33 Tenebrionidae species based on P123 datasets (protein-coding genes [PCGs] with all codon positions) using Bayesian inference (BI). Posterior probabilities for BI are shown on corresponding nodes in the topology of the BI tree. Different colors of species name blocks represent the different species, tribes, and subfamilies.

Three mitogenomic datasets (P123, P123AA, and P123RNA) and two analytical approaches (BI and ML) yielded six phylogenetic trees (Figure 9 and Figure S5), involving 33 tenebrionid species in five subfamilies. Generally, BI trees had higher support values than those of ML trees (Figure 9 and Figure S5). All analyses yielded similar phylogenetic relationships. However, seven species (*Uloma* sp., *U. dermestoides* Chevrolat, *Z. atratus*, *T. molitor*, *B. rhynchoptera*, and two Lagriinae species) showed unstable phylogenetic positions among trees (Figure S5).

Among the five subfamilies, the phylogenies supported the monophyly of Lagriinae (PP = 1, BP > 57), Stenochiinae (PP = 1, BP ≥ 93), and Pimeliinae (PP = 1, BP ≥ 83), while Diaperinae and Tenebrioninae were polyphyletic (Figure 9). At the subfamily level, BI and ML trees based on dataset P123AA were concordant; however, the different positions of *Adelium* sp. and *C. popularis* Borchmann based on datasets P123 and P123RNA (Figure S5) did not support the monophyly of Lagriinae. For the eight tribes within Tenebrioninae, Tenebrionini was not monophyletic. Three phylogenetic groups were obtained for Tenebrioninae: the first included Alphitobiini (*A. diaperinus* Panzer), the second group included Amarygmi (*Amarygmini* sp.) and Ulomini (*Uloma* sp.), and the third group included five tribes was paraphyletic. At the tribe level, Cnodalonini of Stenochiinae (PP = 1, BP ≥ 93), Opatrini (PP = 1, BP = 100), and Triboliini (PP = 1, BP = 100) of Tenebrioninae, and Asidini of Pimiliinae (PP = 1, BP = 100) were monophyletic.

## 4. Discussion

### 4.1. Mitochondrial Genome Organization and Composition

The three newly sequenced mitogenomes of darkling beetles included the typical gene contents reported for other Tenebrionidae mitogenomes. Among the 33 Tenebrionidae species, *P. valgipes* (Marseul) and *C. popularis* both had aberrant AT-skews and GC-skews. The positive GC-skew and negative AT-skew in *P. valgipes* as well as the relatively high GC-skew in *C. popularis* indicated that there was a clear bias towards the utilization of G and C in both species. Ts also occurred more frequently in the backbone than As in both species. In most chains of other insects, there are fewer T than A and fewer G than C bases [43,44], suggesting that the chain asymmetry was reversed [45]. Furthermore, the AT-skew and GC-skew of *P. valgipes* were abnormally positive and negative, suggesting that the chain asymmetry of its mitogenome is reversed.

Several codons with high RSCU values ended in A/T, and codons ending in A/T were used more frequently than those ending in G/C, suggesting a codon bias, as determined by RSCU analyses of 33 Tenebrionidae species. Similar results have been obtained in previous studies [17,46,47]. On the basis of their GC content in relation to their synonyms, codons respond to genome composition according to Robin’s theories of neutral mutations [48]. The A+T content at the third codon position was higher than those of the first and second codons in the mitogenomes of all Tenebrionidae species, indicating that the third codon was more vulnerable to AT alterations [49]. We discovered that species in the subfamily Stenochiinae had lower A+T contents than those of species in other subfamilies; this suggested that these species evolved relatively slowly, in turn suggesting that they showed high phylogenetic stability. This supports the theory that the Stenochiinae subfamily belongs to a stable monophyletic lineage (as described below).

All the 33 Tenebrionidae mitogenomes contained an intergeneric space (15 bp to 19 bp) between *trnS2* (UCN) and *nad1*. Previous studies of tenebrionid mitogenomes had also reported intergenic spacers at this location, with lengths of approximately 17 bp [14,46,50]. Additionally, other Coleoptera families had a similar spacer in this location [51,52]. Li et al. (2007) discovered that the spacer was absent from two members of the genus *Rhagophthalmus* [53]. Insertions and deletions near the end of *nad1* resulting from sequencing errors or correction by posttranslational modification were the cause of this difference [50]. Given that this position denotes the conclusion of the major-strand coding region, it has been hypothesized that this interval may correspond to the binding site of the transcription attenuation factor mtTERM [54]. These intergenic spacer sequences have been shown to be binding sites of a transcription termination factor (DmTTF) [55]. Both *M. exilidentata* and *A. potanini* had a 47 bp spacer between *trnC* and *trnY*; however, this was not a uniform finding, as other species belonging to the same tribe did not have the same spacer. A long intergenic space, measuring 35–376 bp, has been detected between *trnW* and *trnC*; these two tRNAs were transposed and rearranged. This lengthy intergenic region contains tandem repeat units, which are thought to be *trnW* remnants [56]. When comparing the 47 bp sequence to *trnW* of *A. potanini* and *M. exilidentata*, we discovered partial identity. As a result, we assumed that this intergenic region was a remnant of *trnW* post-replication.

With the exception of *trnS1*, the remaining 21 tRNAs had the normal cloverleaf form. Nematode *trnS1* lacks the DHU arm; however, nuclear magnetic resonance studies of its tertiary structure revealed that such an abnormal tRNA may fit the ribosome by altering its structural conformation and function [57]. The tRNA function is ensured by the structural conservation of tRNA in the anticodon and tRNA core region, where the D-loop and T-loop interact to stabilize the overall structure [58].

### 4.2. tRNA-like Structures

We uncovered several tRNA-like structures in the A+T-rich area of 23 darkling beetles, which have never been reported in Tenebrionidae but demonstrated in other Coleoptera [59], Lepidoptera [27], and Hymenoptera [26,28]. Twenty-three out of 33 (69.69%) reported Tenebrionidae mitogenomes had tRNA-like structures, indicating that tRNA-like structures were very common in A+T-rich regions of insects, consistent with a previous study of Lepidoptera [27]. Of note, *trnL2*-like sequences were most frequent, possibly due to the high frequency of usage of the TTA codon encoding leucine. *trnL2*-like sequences may have the highest turnover rate in cells, which may increase the chance of binding to cells [27]. Despite the relatively low usage, the codon that codes for tryptophan has a more conserved sequence, which might explain the high frequency of *trnW*-like sequences in Tenebrionidae mitogenomes. Remarkably, both *trnL2*-like and *trnW*-like sequences were found in three of 32 species. The phylogenetic tree showed that these three species belonged to the same subfamily, Pimeliinae; however, the shared *trnL2*-like and *trnW*-like sequences in these three taxa may not be informative for reconstructing relationships. We analyzed the *trnL2*-like and *trnW*-like sequences of *A. potanini*, *P. contortus* LeConte, and *Pelecyphorus foveolatus* Solier. We discovered that the *trnL2*-like similarity between *A. potanini* and *P. contortus* reached 59.10%, including highly conserved sequences (results not shown). Both *P. contortus* and *Stenomorpha obovate* LeConte, within the same monophyletic lineage, had *trnR*-like and *trnW*-like sequences. Additionally, the two *trnW*-like sequences collectively had multiple conserved sequences of 9–11 bp, which may also explain their grouping into monophyletic lineages. We also analyzed the noncoding regions of *Platydema* sp. and *A. diaperinus*. When the 22 tRNAs of *A. diaperinus* were compared with their A+T-rich regions, there was a highly repetitive fragment with eight tRNA sequences; however, they did not have the same anticodon and therefore we did not identify these as tRNA-like structures. We compared this sequence (74 bp) with the A+T-rich region of *Platydema* sp. and found a high sequence similarity, with 79.73% conservation. The most conserved regions in the A+T-rich regions of *Platydema* sp. and *A. diaperinus* matched the most tRNA-like sequences, which may be related to their evolutionary conservatism, as indicated by present and previous studies which consistently supported a sister relationship between *Platydema* sp. (Diaperinae) and *A. diaperinus* (Tenebrioninae).

In some previous studies, sequences with identical anticodons were regarded as tRNA-like sequences [27,60], while alternative anticodons can be utilized in other studies [26,61]; a consistent standard has not been established [62]. In the tRNA-like analysis in this study, the same anticodon was used as a criterion for identification. Certain appropriate anticodons and secondary structures made A+T-rich sequences partially functional. Some sequences with different anticodons can also form trilobal secondary structures, suggesting that they had unidentified functions. In this regard, Serine transfer between the three tRNA-Ser sequences had been investigated [26]. A *trnN*-like sequence with a different anticodon in *M. unguiculina* had the highest match to the conventional tRNA (72.3%) among tRNAs of all three newly sequenced species. The *trnN*-like sequence was located between in the major-strand *trnI* and minor-strand *rrnS* and overlapped with conventional *trnN* by 47 bp. It had a CCA-OH3′, a TψC arm, an anticodon arm, and a variable arm (Figure S6). We speculated that this *trnN*-like structure had the same function of receiving aspartic acid.

The *trnN*-like ATT anticodon was relatively rare in Tenebrionidae, although it was possible. Anticodon point mutations can lead to codon reassignment [63], and further studies are needed to explore the functional effects of anticodon point mutations on tRNA-like sequences. The pairwise distance between *trnN*-like and regular *trnN* sequences was 0.714 and between *trnN*-like and *trnN* of species in the same tribe (*M. exilidentata*, *O. sabulosum* Linnaeus, and *Gonocephalum* sp.) were 0.714, 0.786, and 0.786, respectively. The pairwise distance between *trnN*-like and *trnN* of the same subfamily was 0.133–0.200, indicating that the regular *trnN* was more closely related to *trnN* of other species (Figure S6). A phylogenetic analysis of *trnN*-like of darkling beetles in the same tribe revealed that regular *trnN* and those of other insects were distantly related to *trnN*-like, forming an independent group (Figure S6).

Possible sources of tRNA-like sequences include strand slippage and mispairing and incorrect replication initiation or termination [64], leading to copy duplication. Mistakes during gene rearrangement can potentially result in unexpected mutations. In studies of mammalian mtDNA heavy strand replication origins, nucleotide sequences that fold into tRNA-like structures may serve as primers for DNA synthesis [65]. For example, tRNAs can function as primers for mtDNA synthesis and integrate a tRNA gene into the mitogenome if the primer is not cleaved from the growing DNA strand [66]. The non-coding A+T-rich region will show increased tRNA-like sequence diversity after insertion. Gene rearrangements, which did not occurred recently in the Tenebrionidae mitogenome, may be involved. As the forward and reverse strands are transcribed separately and the start is in the control region, the tRNA at the intersection should theoretically be closer to the typical structure. Despite the discovery of multiple tRNA-like structures in plant and animal viruses and studies of their essential activities [62], few studies have evaluated their evolutionary characteristics and functions in insect mitogenomes. The conventional and tRNA-like structures differed in sequence homology by 47.14% to 69.12% in this study. All tRNA-like sequences had many stem mismatches. Many studies have found that these tRNA-like structures are not pseudogenes, since they comprise anticodon and clover-leaf structures, which must have similar functional values [26]. Invertebrates have a unique non-coding section called the A+T-rich region that functions similarly to the regulatory region of vertebrate mtDNA [67]. Hence, tRNA-like sequences are common in many insect species, suggesting that the non-coding region may be involved in transcription and termination. Does a better match indicate greater functional effects? Are they still functional? These questions need to be investigated.

### 4.3. Phylogeny of Tenebrionidae

Our mitochondrial phylogeny was generally consistent with traditional morphological classifications and recent molecular studies. However, there were contradictory results for some species depending on the dataset and method of inference, suggesting that mitogenome sequences are still useful genetic markers for resolving molecular phylogenetic relationships at various taxonomic levels in beetles.

At the subfamily level, phylogenies based on different datasets assigned *Adelium* sp. and *C. popularis* to different positions. The monophyly of Lagriinae has been argued in many previous molecular and morphological studies [9,14,16,68]. In this study, based on the dataset P123, Lagriinae was a sister group to the other subfamilies, followed by the subfamily Pimeliinae, in agreement with previous molecular studies [14]. Stenochiinae was part of a clade composed of partial Diaperini (Diaperinae) and Alphitobiini (Tenebrioninae); both Diaperinae and Tenebrioninae were clearly polyphyletic. These results were consistent with some previous molecular studies where Diaperinae was polyphyletic [1,14,17]; however, Diaperinae has been identified as monophyletic based on morphological characters [69]. Furthermore, conflicting results have been obtained based on molecular data; for example, studies have shown that the split between *Platydema* sp. and *U. dermestoides* is more recent than observed in our study [17]. Stenochiinae is a monophyletic group according to morphological studies based on 80 characters [69], consistent with our findings. However, other studies suggested that Stenochiinae belonged to a clade that included partial Diaperini (Diaperinae), Helopinini, and Alphitobini (Tenebrioninae) [14]. Our study supported the monophyly of Pimeliinae, which was in agreement with results of other molecular studies [14]; however, its monophyly was not supported by all previous studies. In morphological studies, Tenebrioninae has been identified as monophyletic, whereas molecular studies supports this group as polyphyly [1,14,16]. This inconsistency may be due to differences in taxa or analytical methods; the dataset is important to phylogenetic tree topology [70].

In the present study, the two species within Diaperini were in different positions on the phylogenetic tree; *U. dermestoides* was sister to *Z.atratus* and *Platydema* sp. was a sister of Alphitobiini in previous studies [71,72]; however, some studies suggested that the two were more distantly related [14,73]. The relationships within Tenebrioninae also varied among three trees constructed using Bayesian methods. In the P123-based tree, Blaptini was in the middle of Tenebrionini, while Diaperinini was more derived than Tenebrionini. In the ML tree, Asidini based on P123AA was sister to *P. contortus*; in the P123-based and P123RNA-based trees, Asidini was a sister group to the monophyletic group formed by *P. contortus* and *P. foveolatus*.

In a morphological analysis based on 80 characters, each of Opatrini, Diaperini, and Tenebrionini within Tenebrioninae was supported as monophyly [69]. The monophyly of Opatrini in Tenebrioninae proposed in this study was well-supported by morphological and molecular data [1,14,16,69], while the monophyly of Diaperini and Tenebrionini is not well-supported by morphological data [69]. Triboliini was monophyletic in the Bayesian tree based on the P123 dataset but not in the ML tree, as reported in previous studies [1,19].

## 5. Conclusions

In this study, we characterized three Tenebrionidae mitogenomes and performed comparative mitogenomic analyses of darkling beetles. The genome size, gene arrangement, base composition, and codon usage were all conserved within the darkling beetles, while diversity in sequence length and structural features was detected in control regions. We presented a brief structural and functional analysis of tRNA-like structures, discussed the possible functions of tRNA-like structures in terms of how well they have been conserved during evolution, and argued that this may be part of the basis for the classification of Tenebrionidae species. This provides some data on the current inadequate tRNA-like studies in the insect mitogenomic field. This study recovered a tenebrionid mitogenomic phylogeny: (Lagriinae, (Pimeliinae, ((Tenebrioninae + Diaperinae), Stenochiinae))), indicating that mitogenomic data could provide useful information for resolving the Tenebrionidae phylogeny. However, additional morphological (more characters) and molecular analyses (more loci) with wider taxonomic sampling are needed for a better understanding of the evolution of darkling beetles.

## Figures and Tables

**Table 1:** Sampling information of the three Tenebrionidae species that were newly sequenced in this study.

Subfamily	Species	Sampling Site	Voucher Specimen Code	Coordinate
Tenebrionidae	*M. exilidentata*	Lingwu County, Ningxia Province, China	YCLW-BJ1	106°34′ E, 38°10′ N
Tenebrionidae	*A. potanini*	Lingwu County, Ningxia Province, China	YCLW-BJ2	106°34′ E, 38°10′ N
Tenebrionidae	*M. unguiculina*	Lingwu County, Ningxia Province, China	YCLW-BJ3	106°34′ E, 38°10′ N

## Data Availability

The data presented in this study are openly available in NCBI (GenBank accession numbers OQ534869, OQ511732, and OQ536315).

## Supplementary Materials

The following supporting information can be downloaded at: https://www.mdpi.com/article/10.3390/genes14091738/s1, Figure S1: Rates of evolution of 13 protein-coding genes in the mitogenomes of 33 Tenebrionidae species. The left Y-axis provides the substitution rate of the mitochondrial gene, while the right Y-axis provides the G+C content. Synonymous nucleotide substitutions per synonymous site (*K_s_*) and nonsynonymous nucleotide substitutions per nonsynonymous site (*K_a_*) are calculated using the Kumar method. The standard errors were obtained by a bootstrap procedure (1000 replicates). Figure S2: *K_a_*/*K_s_* values of the 13 PCGs were negatively correlated with the percentage of GC (*R*^2^ = 0.86, P < 0.01). Figure S3: tRNA-like secondary structures in the A+T-riched regions of 33 Tenebrionidae species with a match of 65% or less. Comparisons with the corresponding conventional tRNA sequences are provided. Orange nucleotides indicate anticodons, which specify the corresponding tRNAs. *, consensus sequences in the alignment. Figure S4: Alignment of the gap region between tRNA-Ser (UCN) and *nad1* in the genomes of all Tenebrionidae species. Colored boxes indicate the conserved nucleotide regions in all beetles, with the highly conserved pentanucleotide region (motif I, motif II, and motif III) in the middle. Figure S5: Phylogenetic relationships among 33 beetles obtained from analyses of PCGs alone using MrBayes (A/C/E) and RAxML (B/D/F). Support values (PP > 0.90; BP > 70) are shown next to nodes in the trees. The scale bar represents substitutions/site. Figure S6: Relationship between *M. unguiculina trnN*, *M. unguiculina* *trnN*-like sequences and representative insect *trnN*. (A) Genetic distances between *M. unguiculina trnN*, *M. unguiculina trnN*-like sequences, and representative insect *trnN*. (B) Phylogenetic relationships among *M. unguiculina trnN*, *M. unguiculina trnN*-like sequences, and representative insect *trnN*. The number on each node indicates the bootstrap support based on 1000 replicates. Table S1: Characteristics of mitogenomes of 33 Tenebrionidae species. Table S2: Saturation test for the 13 protein-coding genes (PCGs, the P123 dataset), concentration of 13 PCGs and two ribosomal RNA genes (the P123RNA dataset), and each of the three positions of 13 PCGs as implemented in DAMBE. Table S3: The best partitioning schemes and substitution models selected by IQ-TREE for the three datasets. Table S4: Annotation and organization of three mitochondrial genomes newly sequenced in this study. Table S5: Codon usage for the 13 mitochondrial protein-coding genes of 33 Tenebrionidae species. n: number of occurrences; %: percentage of total amino acids; RSCU: Relative synonymous codon usage. Table S6: Distribution of tRNA-like sequences in all Tenebrionidae species in this study. Table S7: Number of occurrences and matching of tRNA-like structures in Tenebrionidae mitochondrial genomes.
