# Supplementary material for: Comparative Mitogenomic Analyses of Darkling Beetles (Coleoptera: Tenebrionidae) Provide Evolutionary Insights into tRNA-like Sequences
Source: Genes (Basel). 2023 Aug 30;14(9):1738. doi: 10.3390/genes14091738 (PMC10530909; doi:10.3390/genes14091738)
Supplement: Supplementary file 1 [file genes-14-01738-s001.zip › Figure S4 Non-coding region.pdf]

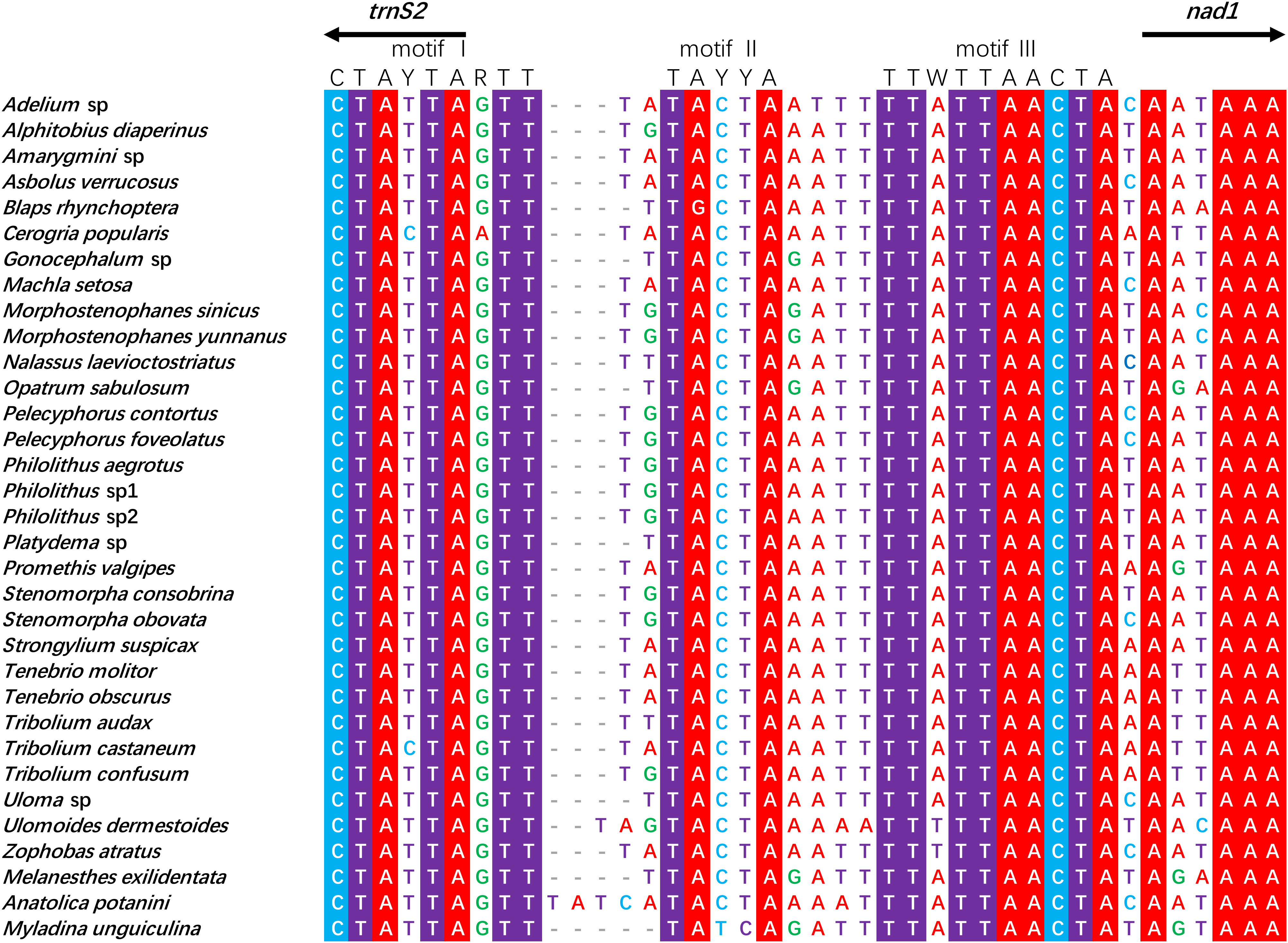
trnS2
nad1
motif I
motif II
motif III
CTAYTARTT TAYYA TTWTTAACTA
Adelium sp CTATTAGTT---TATACTAATTTTTATTAACTACAATAAA
Alphitobius diaperinus CTATTAGTT---TGTACTAAATTTTATTAACTATAATAAA
Amarygmini sp CTATTAGTT---TATACTAAATTTTATTAACTATAATAAA
Asbolus verrucosus CTATTAGTT---TATACTAAATTTTATTAACTACAATAAA
Blaps rhynchoptera CTATTAGTT----TTGCTAAATTTTATTAACTATAAAAAA
Cerogria popularis CTACTAATT---TATACTAAATTTTATTAACTAAATTAAA
Gonocephalum sp CTATTAGTT----TTACTAGATTTTATTAACTATAATAAA
Machla setosa CTATTAGTT---TATACTAAATTTTATTAACTACAATAAA
Morphostenophanes sinicus CTATTAGTT---TGTACTAGATTTTATTAACTATAACAAA
Morphostenophanes yunnanus CTATTAGTT---TGTACTAGATTTTATTAACTATAACAAA
Nalassus laevioctostriatus CTATTAGTT---TTTACTAAATTTTATTAACTACAATAAA
Opatrum sabulosum CTATTAGTT----TTACTAGATTTTATTAACTATAGAAAA
Pelecyphorus contortus CTATTAGTT---TGTACTAAATTTTATTAACTACAATAAA
Pelecyphorus foveolatus CTATTAGTT---TGTACTAAATTTTATTAACTACAATAAA
Philolithus aegrotus CTATTAGTT---TGTACTAAATTTTATTAACTATAATAAA
Philolithus sp1 CTATTAGTT---TGTACTAAATTTTATTAACTATAATAAA
Philolithus sp2 CTATTAGTT---TGTACTAAATTTTATTAACTATAATAAA
Platydema sp CTATTAGTT----TTACTAAATTTTATTAACTATAATAAA
Promethis valgipes CTATTAGTT---TATACTAAATTTTATTAACTAAAGTAAA
Stenomorpha consobrina CTATTAGTT---TGTACTAAATTTTATTAACTATAATAAA
Stenomorpha obovata CTATTAGTT---TGTACTAAATTTTATTAACTACAATAAA
Strongylium suspicax CTATTAGTT---TATACTAAATTTTATTAACTAAAATAAA
Tenebrio molitor CTATTAGTT---TATACTAAATTTTATTAACTAAATTAAA
Tenebrio obscurus CTATTAGTT---TATACTAAATTTTATTAACTAAATTAAA
Tribolium audax CTATTAGTT---TTTACTAAATTTTATTAACTAAATTAAA
Tribolium castaneum CTACTAGTT---TATACTAAATTTTATTAACTAAATTAAA
Tribolium confusum CTATTAGTT---TGTACTAAATTTTATTAACTAAATTAAA
Uloma sp CTATTAGTT----TTACTAAATTTTATTAACTACAATAAA
Ulomoides dermestoides CTATTAGTT--TAGTACTAAAAATTTTTAACTATAACAAA
Zophobas atratus CTATTAGTT---TATACTAAATTTTTTTAACTACAATAAA
Melanesthes exilidentata CTATTAGTT----TTACTAGATTTTATTAACTATAGAAAA
Anatolica potanini CTATTAGTTTATCATACTAAAATTTATTAACTACAATAAA
Myladina unguiculina CTATTAGTT-----TATCAGATTTTATTAACTATAGTAAA
