# Supplementary material for: Comparative Mitogenomic Analyses of Darkling Beetles (Coleoptera: Tenebrionidae) Provide Evolutionary Insights into tRNA-like Sequences
Source: Genes (Basel). 2023 Aug 30;14(9):1738. doi: 10.3390/genes14091738 (PMC10530909; doi:10.3390/genes14091738)

A

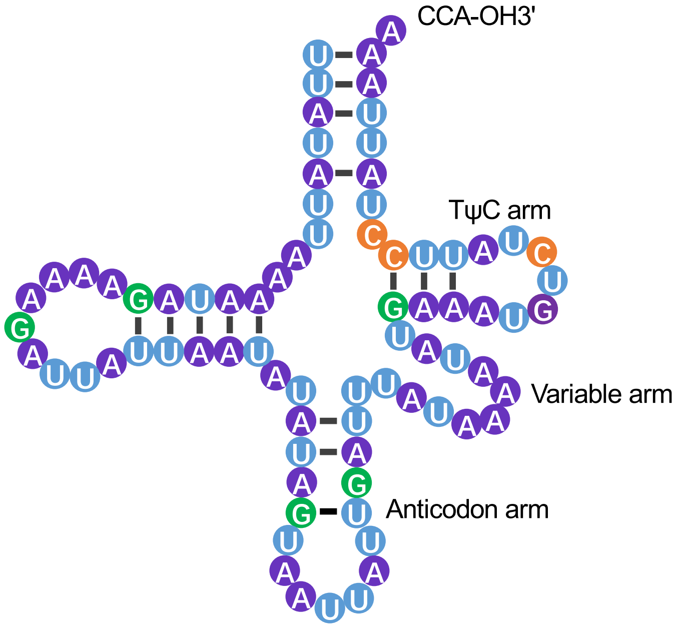

trnN-like
Myladina unguiculina
72.30%

B

		1	2	3	4	5	6	7	8	9	10	11	12	13	14
1	*Gonocephalum* sp *trnN*														
2	*Opatrum sabulosum trnN*	0.133													
3	*Melanesthes exilidentata trnN*	0.067	0.067												
4	*Myladina unguiculina trnN*	0.133	0.067	0.133											
5	*Tenebrio molitor trnN*	0.067	0.200	0.133	0.200										
6	*Tenebrio obscurus trnN*	0.133	0.133	0.133	0.067	0.200									
7	*Blaps rhynchoptera trnN*	0.067	0.067	0.000	0.133	0.133	0.133								
8	*Tribolium audax trnN*	0.133	0.200	0.133	0.133	0.200	0.133	0.133							
9	*Tribolium castaneum trnN*	0.133	0.067	0.133	0.000	0.200	0.067	0.133	0.133						
10	*Tribolium confusum trnN*	0.133	0.067	0.133	0.000	0.200	0.067	0.133	0.133	0.000					
11	*Myladina unguiculina trnN*-like	0.786	0.714	0.714	0.786	0.786	0.786	0.714	0.786	0.786	0.786				
12	*Melanesthes exilidentata trnN*-like	0.786	0.714	0.714	0.786	0.786	0.786	0.714	0.786	0.786	0.786	0.071			
13	*Gonocephalum* sp *trnN*-like	0.733	0.733	0.733	0.667	0.733	0.667	0.733	0.667	0.667	0.667	0.857	0.857		
14	*Opatrum sabulosum trnN*-like	0.867	0.800	0.867	0.800	0.933	0.867	0.867	0.867	0.800	0.800	0.786	0.714	0.867	

C

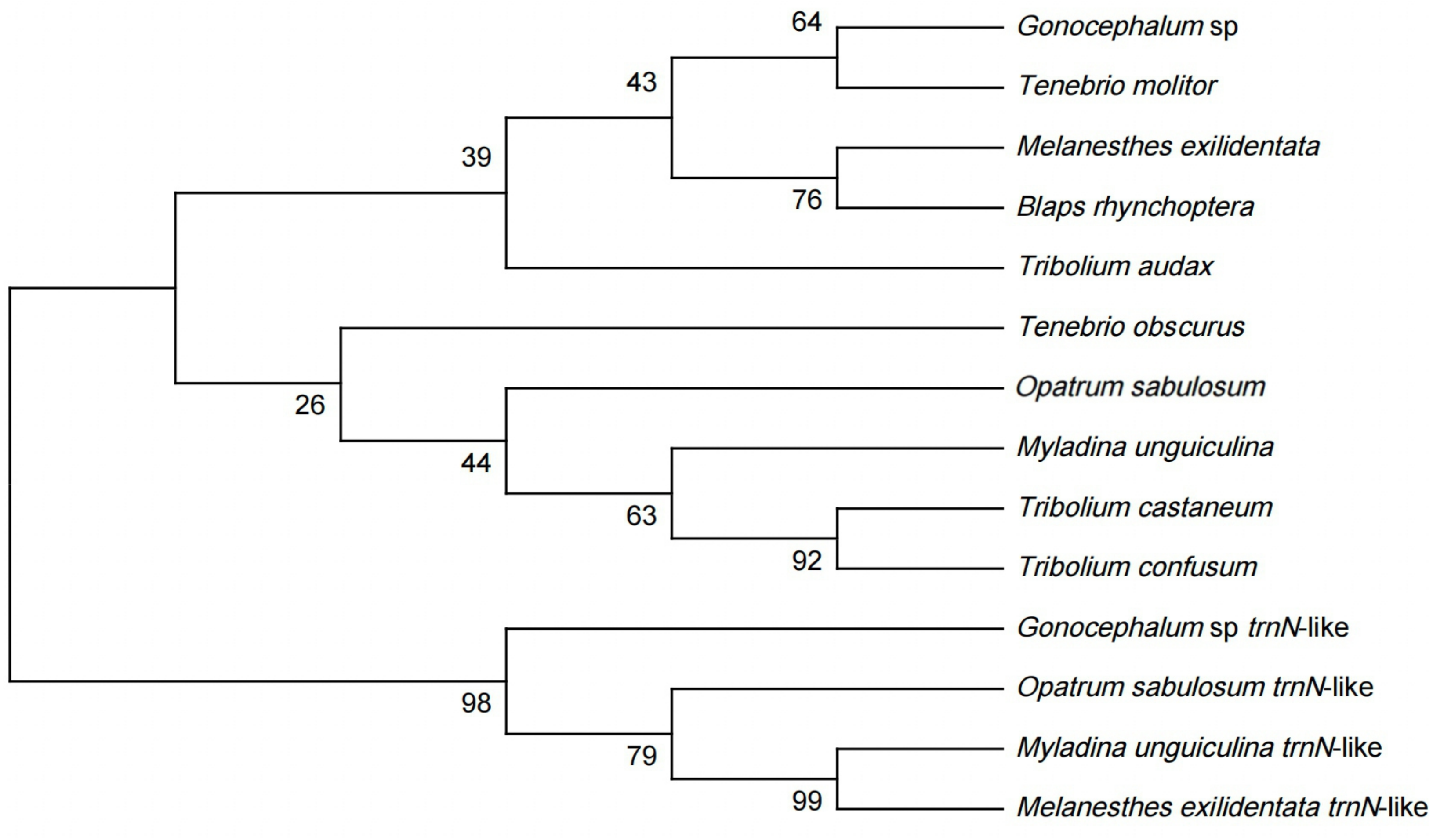

Supplement: Supplementary file 1 [file genes-14-01738-s001.zip › Figure S6 Relationship trnN-trnN-like sequences.pdf]
