# Supplementary material for: Comparative Mitogenomic Analyses of Darkling Beetles (Coleoptera: Tenebrionidae) Provide Evolutionary Insights into tRNA-like Sequences
Source: Genes (Basel). 2023 Aug 30;14(9):1738. doi: 10.3390/genes14091738 (PMC10530909; doi:10.3390/genes14091738)
Supplement: Supplementary file 1 [file genes-14-01738-s001.zip › Table S1 Characteristics of mitogenomes of 33 Tenebrionidae species.pdf]

Table S1 Characteristics of mitogenomes of 33 Tenebrionidae species.

Famliy	Subfamliy	Species	GenBank accession number	Genome Size (bp)
Tenebrionidae	Lagriinae	*Adelium* sp	NC_013554	16449
Tenebrionidae	Alphitobius	*Alphitobius diaperinus*	NC_049092	15511
Tenebrionidae	unclassified Tenebrioninae.	*Amarygmini* sp	MH789725	15942
Tenebrionidae	Pimeliinae	*Asbolus verrucosus*	NC_027256	15828
Tenebrionidae	Blaptinae	*Blaps rhynchoptera*	NC_047449	16149
Tenebrionidae	Lagriinae	*Cerogria popularis*	NC_061196	16175
Tenebrionidae	Blaptinae	*Gonocephalum* sp	NC_053250	15836
Tenebrionidae	Pimeliinae	*Machla setosa*	MZ342779	16081
Tenebrionidae	Stenochiinae	*Morphostenophanes sinicus*	MW853764	15662
Tenebrionidae	Stenochiinae	*Morphostenophanes yunnanus*	MZ298928	15690
Tenebrionidae	Tenebrioninae	*Nalassus laevioctostriatus*	KT876905	15529
Tenebrionidae	Blaptinae	*Opatrum sabulosum*	NC_057274	16079
Tenebrionidae	Pimeliinae	*Pelecyphorus contortus*	MZ342780	16244
Tenebrionidae	Pimeliinae	*Pelecyphorus foveolatus*	MZ342781	16218
Tenebrionidae	Pimeliinae	*Philolithus aegrotus*	MZ342784	16268
Tenebrionidae	Pimeliinae	*Philolithus* sp1	MZ342776	15927
Tenebrionidae	Pimeliinae	*Philolithus* sp2	MZ342777	15926
Tenebrionidae	Diaperinae	*Platydema* sp	JX412842	16028
Tenebrionidae	Stenochiinae	*Promethis valgipes*	NC_054362	15801
Tenebrionidae	Pimeliinae	*Stenomorpha consobrina*	MZ342785	16243
Tenebrionidae	Pimeliinae	*Stenomorpha obovata*	MZ342786	16223
Tenebrionidae	Stenochiinae	*Strongylium suspicax*	JX412780	12561

Tenebrionidae	Tenebrioninae	*Tenebrio molitor*	NC_024633	15785
Tenebrionidae	Tenebrioninae	*Tenebrio obscurus*	NC_037196	15771
Tenebrionidae	Tenebrionidae incertae sedis	*Tribolium audax*	NC_024600	15925
Tenebrionidae	Tenebrionidae incertae sedis	*Tribolium castaneum*	NC_003081	15881
Tenebrionidae	Tenebrionidae incertae sedis	*Tribolium confusum*	NC_026702	15813
Tenebrionidae	Tenebrioninae	*Uloma* sp	KT876915	15909
Tenebrionidae	Diaperinae	*Ulomoides dermestoides*	NC_025332	15434
Tenebrionidae	Tenebrioninae	*Zophobas atratus*	NC_041101	15494
**Tenebrionidae**	**Tenebrioninae**	***Melanesthes exilidentata***	**OQ_534869**	**15804**
**Tenebrionidae**	**Pimeliinae**	***Anatolica potanini***	**OQ_511732**	**15809**
**Tenebrionidae**	**Tenebrioninae**	***Myladina unguiculina***	**OQ_536315**	**15542**
