# Supplementary material for: Comparative Mitogenomic Analyses of Darkling Beetles (Coleoptera: Tenebrionidae) Provide Evolutionary Insights into tRNA-like Sequences
Source: Genes (Basel). 2023 Aug 30;14(9):1738. doi: 10.3390/genes14091738 (PMC10530909; doi:10.3390/genes14091738)
Supplement: Supplementary file 1 [file genes-14-01738-s001.zip › Table S2 Saturation Test by DAMBE.pdf]

Table S2 Saturation test for the 13 protein-coding genes (PCGs, the P123 dataset), concentration of 13 PCGs and two ribosomal RNA genes (the P123RNA dataset), and each of the three positions of 13 PCGs as implemented in DAMBE.

	Dataset	P123	P123RNA	Position 1st	Position 2nd	Position 3rd
	Gene size (nt)	11171	13378	3723	3723	3723
NumOTU-4	Iss	0.343	0.366	0.268	0.135	0.137
	Iss.c	0.858	0.859	0.849	0.849	0.849
	P	0	0	0	0	0
NumOTU-8	Iss	0.349	0.367	0.265	0.139	0.139
	Iss.c	0.845	0.844	0.842	0.842	0.842
	P	0	0	0	0	0
NumOTU-16	Iss	0.349	0.383	0.273	0.145	0.14
	Iss.c	0.85	0.853	0.827	0.827	0.827
	P	0	0	0	0	0
NumOTU-32	Iss	0.356	0.395	0.282	0.149	0.149
	Iss.c	0.818	0.819	0.809	0.809	0.809
	P	0	0	0	0	0
NumOTU-4	Iss	0.343	0.366	0.268	0.135	0.137
	Iss.cA	0.846	0.847	0.837	0.837	0.837
	P	0	0	0	0	0
NumOTU-8	Iss	0.349	0.367	0.265	0.139	0.139
	Iss.cA	0.762	0.761	0.758	0.758	0.758
	P	0	0	0	0	0
NumOTU-16	Iss	0.349	0.383	0.273	0.145	0.14
	Iss.cA	0.676	0.675	0.668	0.668	0.668
	P	0	0	0	0	0
NumOTU-32	Iss	0.356	0.395	0.282	0.149	0.149
	Iss.cA	0.572	0.573	0.809	0.554	0.554
	P	0	0	0	0	0
