# Supplementary material for: Comparative Mitogenomic Analyses of Darkling Beetles (Coleoptera: Tenebrionidae) Provide Evolutionary Insights into tRNA-like Sequences
Source: Genes (Basel). 2023 Aug 30;14(9):1738. doi: 10.3390/genes14091738 (PMC10530909; doi:10.3390/genes14091738)
Supplement: Supplementary file 1 [file genes-14-01738-s001.zip › Table S3 The best partitioning schemes.pdf]

Table S3 The best partitioning schemes and substitution models selected by IQ-TREE for the three datasets.

Dataset	Subset	Best-fit scheme	Model
P123	P1	*atp6*_pos1+*cob*_pos1+*cox1*_pos1+*cox2*_pos1+*cox3*_pos1	GTR+F+I+G4
	P2	*atp6*_pos2+*cob*_pos2+*cox1*_pos2+*cox2*_pos2+*cox3*_pos2+*nad1*_pos2+*nad2*_pos2+*nad3*_pos2 +*nad4*_pos2+*nad4L*_pos2+*nad5*_pos2+*nad6*_pos2	TVM+F+I+G4
	P3	*atp6*_pos3+*atp8*_pos3+*cob*_pos3+*cox1*_pos3+*cox2*_pos3+*cox3*_pos3+*nad2*_pos3+*nad3*_pos 3+*nad6*_pos3	TPM3+F+I+G4
	P4	*atp8*_pos1+*atp8*_pos2+*nad2*_pos1+*nad3*_pos1+*nad6*_pos1	GTR+F+I+G4
	P5	*nad1*_pos1+*nad4*_pos1+*nad4L*_pos1+*nad5*_pos1	GTR+F+I+G4
	P6	*nad1*_pos3+*nad4*_pos3+*nad4L*_pos3+*nad5*_pos3	K3Pu+F+I+G4
P123RNA	P1	*atp6*_pos1+*cob*_pos1+*cox1*_pos1+*cox2*_pos1+*cox3*_pos1	GTR+F+I+G4
	P2	*atp6*_pos2+*cob*_pos2+*cox1*_pos2+*cox2*_pos2+*cox3*_pos2+*nad1*_pos2+*nad2*_pos2+*nad3*_pos2 +*nad4*_pos2+*nad4L*_pos2+*nad5*_pos2+*nad6*_pos2	TVM+F+I+G4
	P3	*atp6*_pos3+*atp8*_pos3+*cob*_pos3+*cox1*_pos3+*cox2*_pos3+*cox3*_pos3+*nad2*_pos3+*nad3*_pos 3+*nad6*_pos3	TPM3+F+I+G4
	P4	*atp8*_pos1+*atp8*_pos2+*nad2*_pos1+*nad3*_pos1+*nad6*_pos1	GTR+F+I+G4
	P5	*nad1*_pos1+*nad4*_pos1+*nad4L*_pos1+*nad5*_pos1+*rrnL*+*rrnS*	TVM+F+I+G4
	P6	*nad1*_pos3+*nad4*_pos3+*nad4L*_pos3+*nad5*_pos3	K3Pu+F+I+G4
P123AA	P1	*atp6*+*atp8*+*nad2*+*nad3*+*nad6*	mtMet+F+I+G4
	P2	*cob*+*cox1*+*cox2*+*cox3*	mtART+I+G4
	P3	*nad1*+*nad4*+*nad4L*+*nad5*	mtZOA+F+I+G4
