# Supplementary material for: Comparative Mitogenomic Analyses of Darkling Beetles (Coleoptera: Tenebrionidae) Provide Evolutionary Insights into tRNA-like Sequences
Source: Genes (Basel). 2023 Aug 30;14(9):1738. doi: 10.3390/genes14091738 (PMC10530909; doi:10.3390/genes14091738)
Supplement: Supplementary file 1 [file genes-14-01738-s001.zip › Table S4 Annotation and organization of the complete mitochondrial genome .pdf]

Table S4 Annotation and organization of three mitochondrial genomes newly sequenced in this study.

Annotation and organization of the complete mitochondrial genome of *Melanesthes exilidentata*								
Gene	Strand	Start	End	Length (bp)	Intergenic nucleotides	Start codon	Stop codon	Anticodon
cox1	J	1	1542	1542	0	TTA	TAA	
trnL2	J	1537	1603	67	-6			TAA
cox2	J	1604	2291	688	0	ATA	T	
trnK	J	2292	2362	71	0			CTT
trnD	J	2362	2427	66	-1			GTC
atp8	J	2428	2586	159	0	ATC	TAG	
atp6	J	2580	3251	672	-7	ATG	TAA	
cox3	J	3251	4035	785	-1	ATG	TA	
trnG	J	4035	4097	63	-1			TCC
nad3	J	4098	4451	354	0	ATT	TAG	
trnA	J	4451	4515	65	-1			TGC
trnR	J	4515	4579	65	-1			TCG
trnN	J	4579	4645	67	-1			GTT
trnS1	J	4646	4706	61	0			TCT
trnE	J	4707	4768	62	0			TTC
trnF	N	4767	4831	65	-2			GAA
nad5	N	4835	6545	1711	3	ATT	T	
trnH	N	6546	6609	64	0			GTG
nad4	N	6610	7942	1333	0	ATG	T	
nad4L	N	7936	8217	282	-7	ATA	TAA	
trnT	J	8225	8289	65	7			TGT
trnP	N	8290	8355	66	0			TGG
nad6	J	8358	8852	495	2	ATT	TAA	
cob	J	8852	9988	1137	-1	ATG	TAA	
trnS2	J	9987	10054	68	-2			TGA
nad1	N	10071	11021	951	16	TTG	TAG	
trnL1	N	11022	11087	66	0			TAG
rrnL	N	11091	12367	1277	3			
trnV	N	12368	12436	69	0			TAC
rrnS	N	12437	13204	768	0			
CR	J	13205	14358	1154	0			
trnI	J	14359	14422	64	-3			GAT
trnQ	N	14420	14488	69	-2			TTG
trnM	J	14487	14556	70	0			CAT
nad2	J	14557	15567	1011	0	ATT	TAG	
trnW	J	15571	15636	66	3			TCA
trnC	N	15684	15744	61	47			GCA
trnY	N	15745	5	65	0			GTA

Annotation and organization of the complete mitochondrial genome of *Anatolica potanini*

Gene	Strand	Start	End	Length (bp)	Intergenic nucleotides	Start codon	Stop codon	Anticodon
cox1	J	1	1537	1540	0	ACG	T	
trnL2	J	1538	1603	65	0			TAA
cox2	J	1612	2284	688	0	ATG	T	
trnK	J	2292	2362	71	0			CTT
trnD	J	2362	2427	66	-1			GTC
atp8	J	2428	2586	159	0	ATT	TAG	
atp6	J	2580	3251	672	-7	ATG	TAA	
cox3	J	3251	4034	785	-1	ATG	T	
trnG	J	4035	4097	63	-1			TCC
nad3	J	4098	4451	354	-2	ATT	TAG	
trnA	J	4449	4515	67	-3			TGC
trnR	J	4515	4580	66	-1			TCG
trnN	J	4580	4646	67	-1			GTT
trnS1	J	4647	4707	61	0			TCT
trnE	J	4708	4770	63	0			TTC
trnF	N	4769	4833	65	-2			GAA
nad5	N	4837	6544	1708	3	ATT	T	
trnH	N	6545	6611	67	0			GTG
nad4	N	6612	7944	1333	0	ATG	T	
nad4L	N	7938	8219	282	-7	ATA	TAA	
trnT	J	8228	8291	64	8			TGT
trnP	N	8292	8357	66	0			TGG
nad6	J	8360	8854	495	2	ATT	TAA	
cob	J	8854	9990	1137	-1	ATG	TAA	
trnS2	J	9989	10056	68	-2			TGA
nad1	N	10073	11023	951	16	TTG	TAG	
trnL1	N	11024	11088	65	0			TAG
rrnL	N	11092	12369	1278	3			
trnV	N	12370	12438	69	0			TAC
rrnS	N	12439	13204	766	0			
CR	J	13205	14358	1154	0			
trnI	J	14359	14422	64	0			GAT
trnQ	N	14420	14488	69	-3			TTG
trnM	J	14488	14556	69	-1			CAT
nad2	J	14557	15567	1011	0	ATT	TAG	
trnW	J	15571	15636	66	3			TCA
trnC	N	15684	15744	61	47			GCA
trnY	N	15745	15809	65	0			GTA

Annotation and organization of the complete mitochondrial genome of *Myladina unguiculina*

Gene	Strand	Start	End	Length (bp)	Intergenic nucleotides	Start codon	Stop codon	Anticodon
cox1	J	1	1540	1540	0	ATT	TAA	
trnL2	J	1541	1605	65	0			TAA
cox2	J	1606	2293	688	0	ATC	T	
trnK	J	2294	2364	71	0			CTT
trnD	J	2365	2429	65	0			GTC
atp8	J	2430	2588	159	0	ATA	TAG	
atp6	J	2582	3253	672	-7	ATG	TAA	
cox3	J	3253	4037	785	-1	ATG	TA	
trnG	J	4037	4099	63	-1			TCC
nad3	J	4100	4453	354	0	ATA	TAG	
trnA	J	4451	4518	68	-3			TGC
trnR	J	4516	4579	64	-3			TCG
trnN	J	4580	4644	65	0			GTT
trnS1	J	4645	4705	61	0			TCT
trnE	J	4706	4767	62	0			TTC
trnF	N	4766	4830	65	-2			GAA
nad5	N	4831	6522	1692	0	ATT	TAG	
trnH	N	6542	6605	64	19			GTG
nad4	N	6612	7938	1327	6	ATG	T	
nad4L	N	7931	8219	289	-8	ATG	TAA	
trnT	J	8222	8286	65	2			TGT
trnP	N	8287	8352	66	0			TGG
nad6	J	8355	8849	495	2	ATA	TAA	
cob	J	8849	9988	1140	-1	ATG	TAA	
trnS2	J	9987	10053	67	-2			TGA
nad1	N	10074	11024	951	20	CTG	TAG	
trnL1	N	11025	11087	63	0			TAG
rrnL	N	11088	12368	1281	0			
trnV	N	12369	12438	70	0			TAC
rrnS	N	12439	13203	765	0			
CR	J	13204	14133	930	0			
trnI	J	14134	14197	64	0			GAT
trnQ	N	14195	14263	69	-3			TTG
trnM	J	14263	14332	70	-1			CAT
nad2	J	14332	15342	1011	-1	ATT	TAA	
trnW	J	15341	15408	68	-2			TCA
trnC	N	15418	15478	61	9			GCA
trnY	N	15479	8	64	0			GTA
