# Supplementary material for: Comparative Mitogenomic Analyses of Darkling Beetles (Coleoptera: Tenebrionidae) Provide Evolutionary Insights into tRNA-like Sequences
Source: Genes (Basel). 2023 Aug 30;14(9):1738. doi: 10.3390/genes14091738 (PMC10530909; doi:10.3390/genes14091738)
Supplement: Supplementary file 1 [file genes-14-01738-s001.zip › Table S6 Distribution of tRNA-like sequences of 33 Tenebrionidae species.pdf]

Table S6 Distribution of tRNA-like sequences in all Tenebrionidae species in this study.

Species	tRNA-like	Matching (%)	tRNA-like	Matching (%)	tRNA-like	Matching (%)
Adelium sp	*trnD*-like	65.63	*trnN*-like	59.10		
Alphitobius diaperinus	*trnF*-like	62.12	*trnL2*-like	52.94	*trnP*-like	60.61
Amarygmini sp	*trnE*-like	61.90	*trnL2*-like	52.94		
Blaps rhynchoptera	*trnE*-like	69.12	*trnT*-like	63.08		
Gonocephalum sp	*trnV*-like	58.57	*trnW*-like	58.21		
Machla setosa	*trnK*-like	64.79	*trnS2*-like	58.82	*trnV*-like	59.10
Morphostenophanes sinicus	*trnL2*-like	55.22				
Nalassus laevioctriatus	*trnS2*-like	67.75	*trnW*-like	60.30		
Pelecyphorus contortus	*trnL2*-like	53.85	*trnR*-like	56.10	*trnW*-like	56.10
Pelecyphorus foveolatus	*trnL2*-like	57.58	*trnW*-like	54.55		
Philolithus aegrotus	*trnI*-like	57.81				
Promethis valgipes	*trnL2*-like	53.03	*trnM*-like	47.14		
Stenomorpha consobrina	*trnV*-like	62.86				
Stenomorpha obovata	*trnW*-like	54.55	*trnN*-like	53.85	*trnR*-like	60.61
Tenebrio obscurus	*trnL2*-like	49.25	*trnV*-like	55.71		
Tribolium audax	*trnC*-like	58.06	*trnE*-like	66.15		
Tribolium castaneum	*trnS1*-like	65.00				
Tribolium confusum	*trnL1*-like	55.88	*trnT*-like	57.81	*trnW*-like	58.46
Uloma sp	*trnW*-like	56.72				
Ulomoides_dermestoides	*trnL2*-like	52.31	*trnS1*-like	68.33	*trnY*-like	54.69
Melanesthes exilidentata	*trnI*-like	57.81				
Anatolica potanini	*trnL2*-like	54.55	*trnK*-like	50.70	*trnW*-like	59.10
Myladina unguiculina	*trnE*-like	66.13	*trnV*-like	58.57		
