# Supplementary material for: Comparative Mitogenomic Analyses of Darkling Beetles (Coleoptera: Tenebrionidae) Provide Evolutionary Insights into tRNA-like Sequences
Source: Genes (Basel). 2023 Aug 30;14(9):1738. doi: 10.3390/genes14091738 (PMC10530909; doi:10.3390/genes14091738)
Supplement: Supplementary file 1 [file genes-14-01738-s001.zip › Table S7 Number of occurrences and matching of tRNA.pdf]

Table S7 Number of occurrences and matching of tRNA-like structures in Tenebrionidae mitochondrial genomes.

tRNA-like	Times	Matching Range (%)	Presence In Species
trnC-like	1	58.06	*Tribolium audax*
trnD-like	1	65.63	*Adelium* sp
trnE-like	4	61.90~69.12	*Amarygmini* sp, *Blaps rhynchoptera*, *Myladina unguiculina*, *Tribolium audax*
trnF-like	1	62.12	*Alphitobius diaperinus*
trnI-like	2	57.81	*Melanesthes exilidentata*, *Philolithus aegrotus*
trnK-like	2	50.70~64.79	*Anatolica potanini*, *Machla setosa*
trnL1-like	1	55.88	*Tribolium confusum*
trnL2-like	9	49.25~57.58	*Anatolica potanini*, *Alphitobius diaperinus*, *Amarygmini* sp, *Morphostenophanes sinicus*, *Pelecyphorus foveolatus*, *Pelecyphorus contortus* *Promethis valgipes*, *Tenebrio obscurus*, *Ulomoides dermestoides*
trnM-like	1	47.14	*Promethis valgipes*
trnN-like	2	53.85~59.10	*Adelium* sp, *Stenomorpha obovata*
trnP-like	1	60.61	*Alphitobius diaperinus*
trnR-like	2	56.10~60.61	*Pelecyphorus contortus*, *Stenomorpha obovata*
trnS1-like	2	65.00~68.33	*Tribolium castaneum*, *Ulomoides dermestoides*
trnS2-like	2	58.82~67.75	*Machla setosa*, *Nalassus laevioctriatus*
trnT-like	1	63.08	*Tribolium confusum*
trnV-like	5	55.71~62.86	*Gonocephalum* sp, *Machla setosa*, *Myladina unguiculina*, *Stenomorpha consobrina*, *Tenebrio obscurus*
trnW-like	7	54.55~59.10	*Anatolica potanini*, *Gonocephalum* sp, *Nalassus laevioctriatus*, *Pelecyphorus contortus*, *Pelecyphorus foveolatus*, *Stenomorpha obovate*, *Tribolium confusum*, *Uloma* sp
trnY-like	1	54.69	*Ulomoides dermestoides*
