# Supplementary figures and images for: Comparative Mitogenomic Analyses of Darkling Beetles (Coleoptera: Tenebrionidae) Provide Evolutionary Insights into tRNA-like Sequences
Source: Genes (Basel). 2023 Aug 30;14(9):1738. doi: 10.3390/genes14091738 (PMC10530909; doi:10.3390/genes14091738)

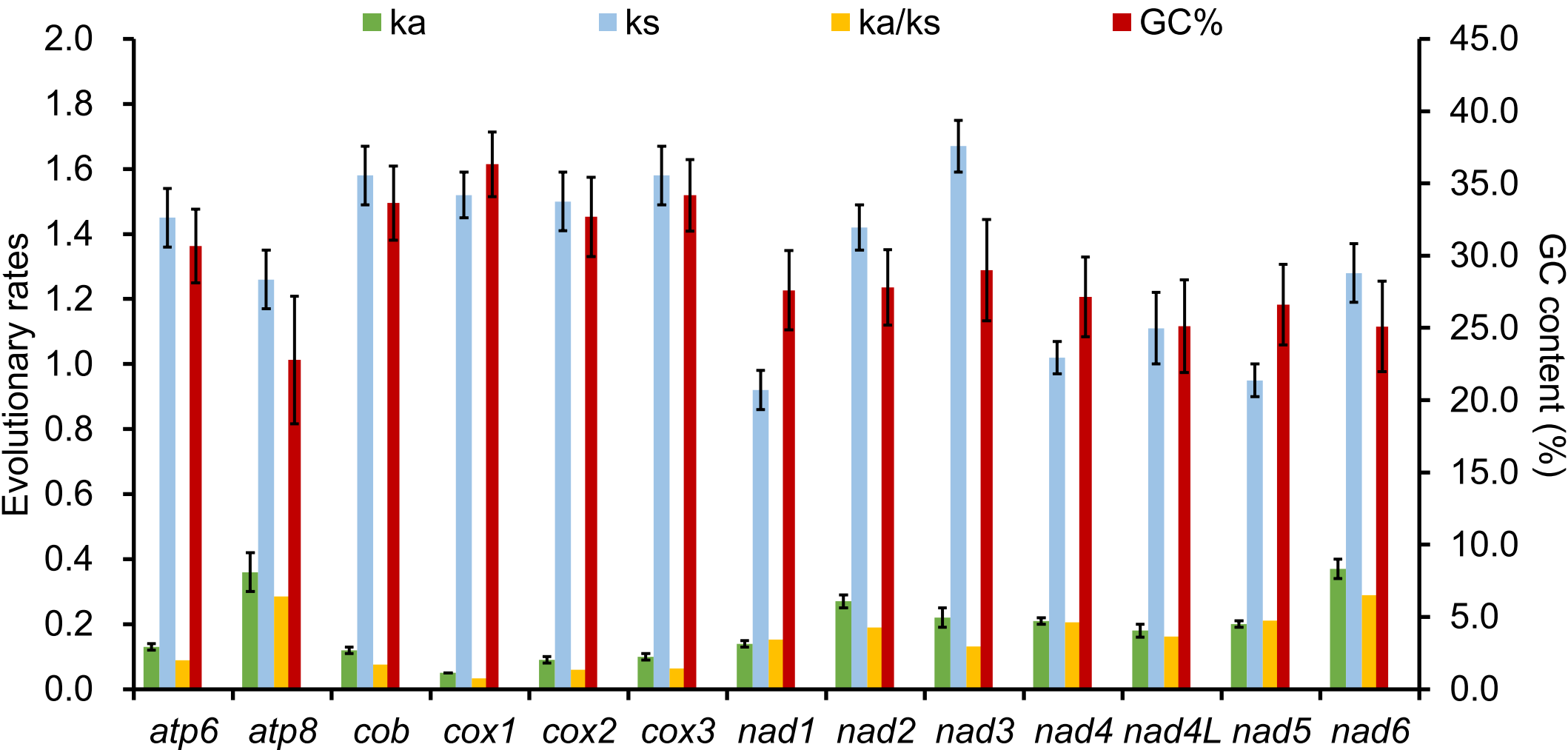

Supplement: Supplementary file 1 [file genes-14-01738-s001.zip › Figure S1 Evolutionary rates of 13 protein-coding.pdf]

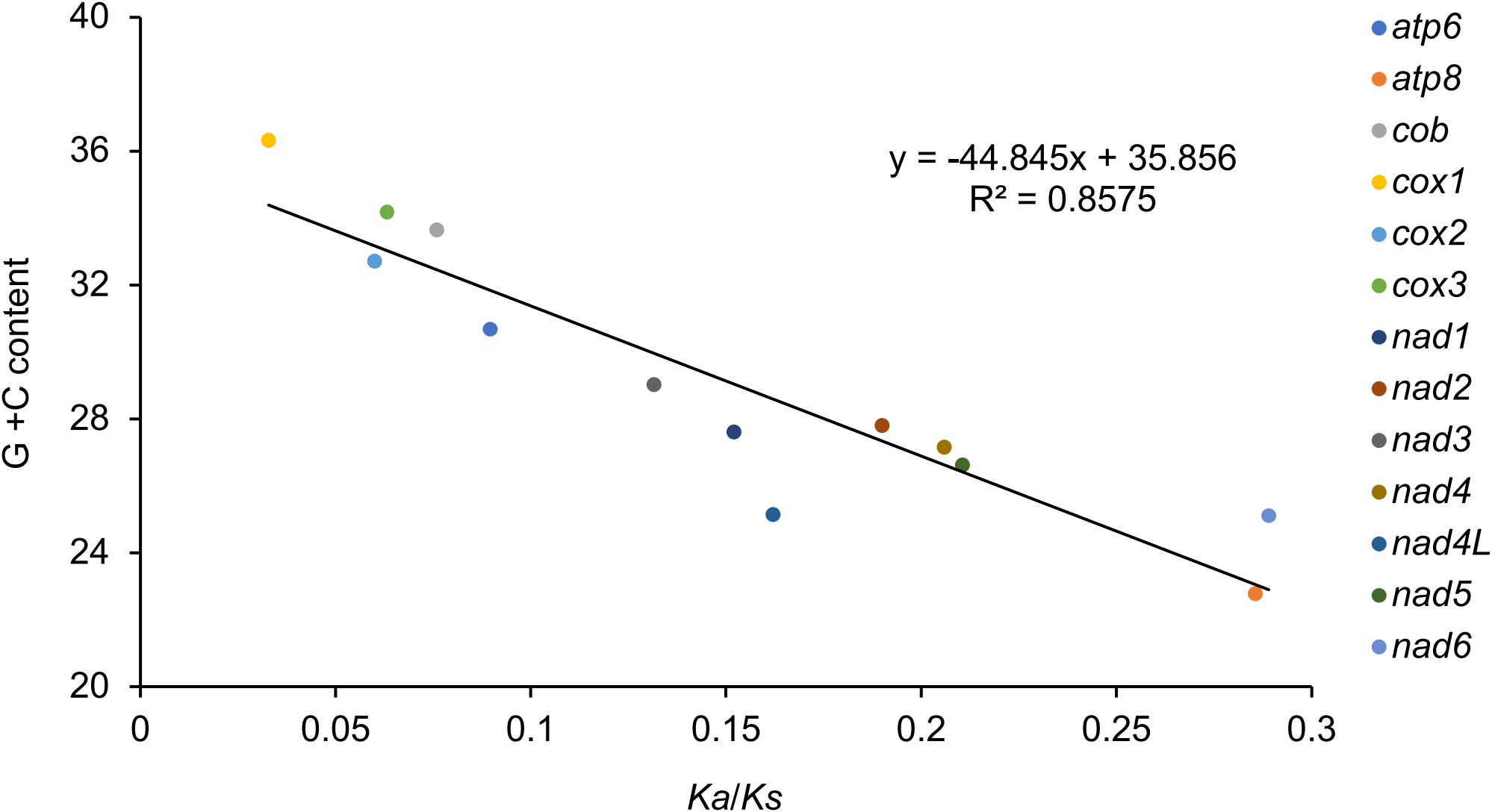

Supplement: Supplementary file 1 [file genes-14-01738-s001.zip › Figure S2 Evolutionary rates of G+C content.pdf]

A MrBayes\_P123

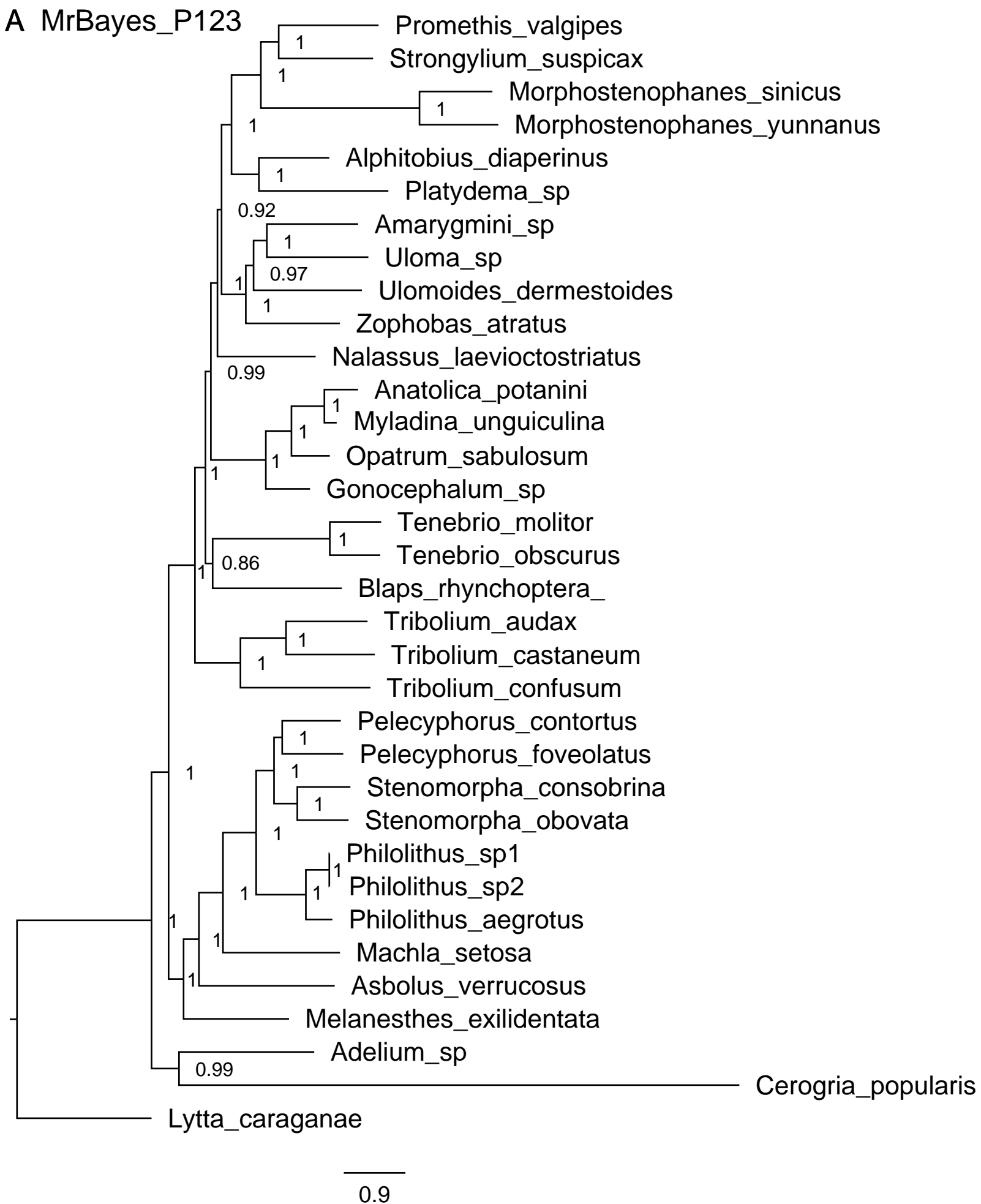

B RAXML\_P123

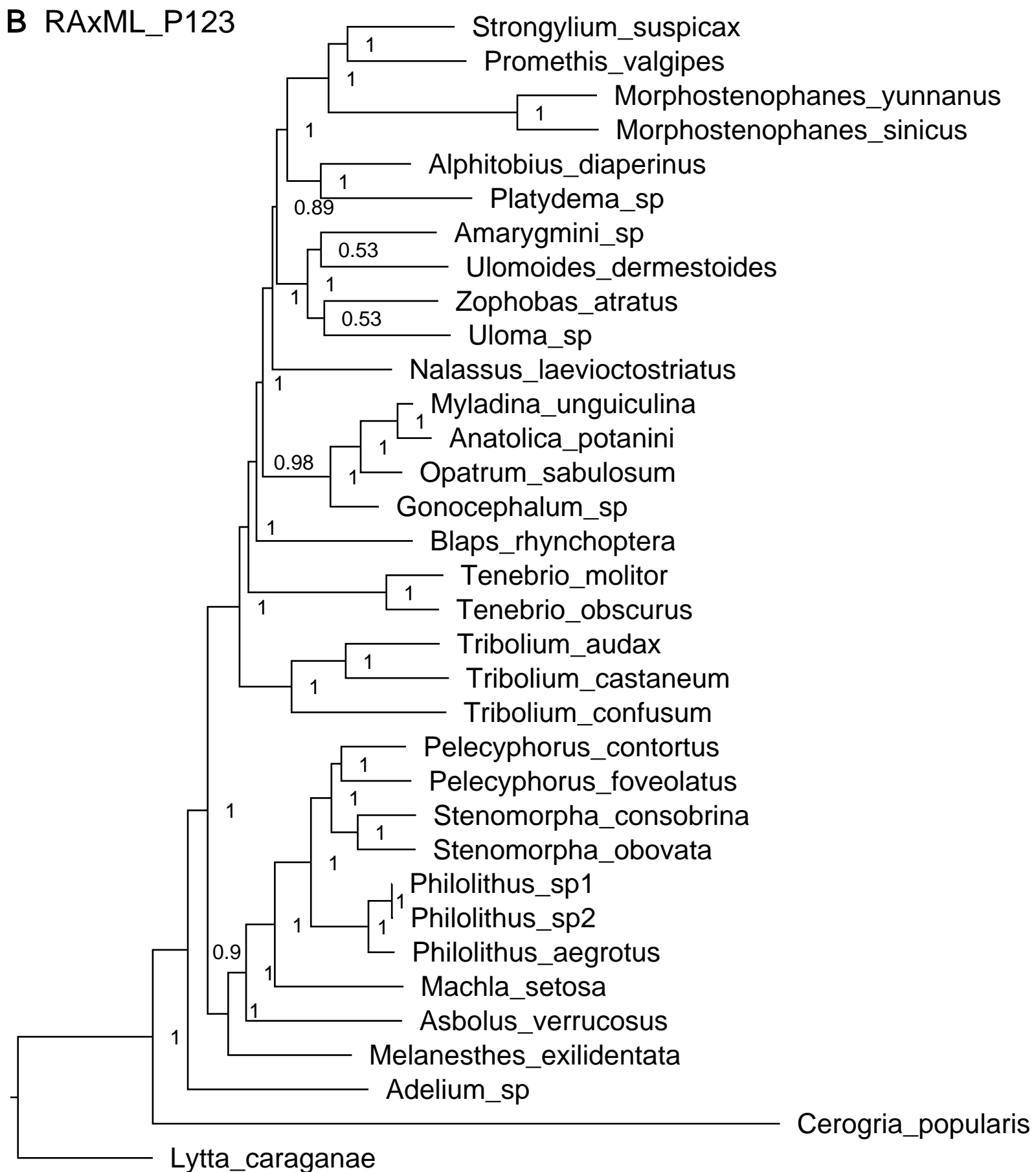

0.7

C MrBayes\_P123AA

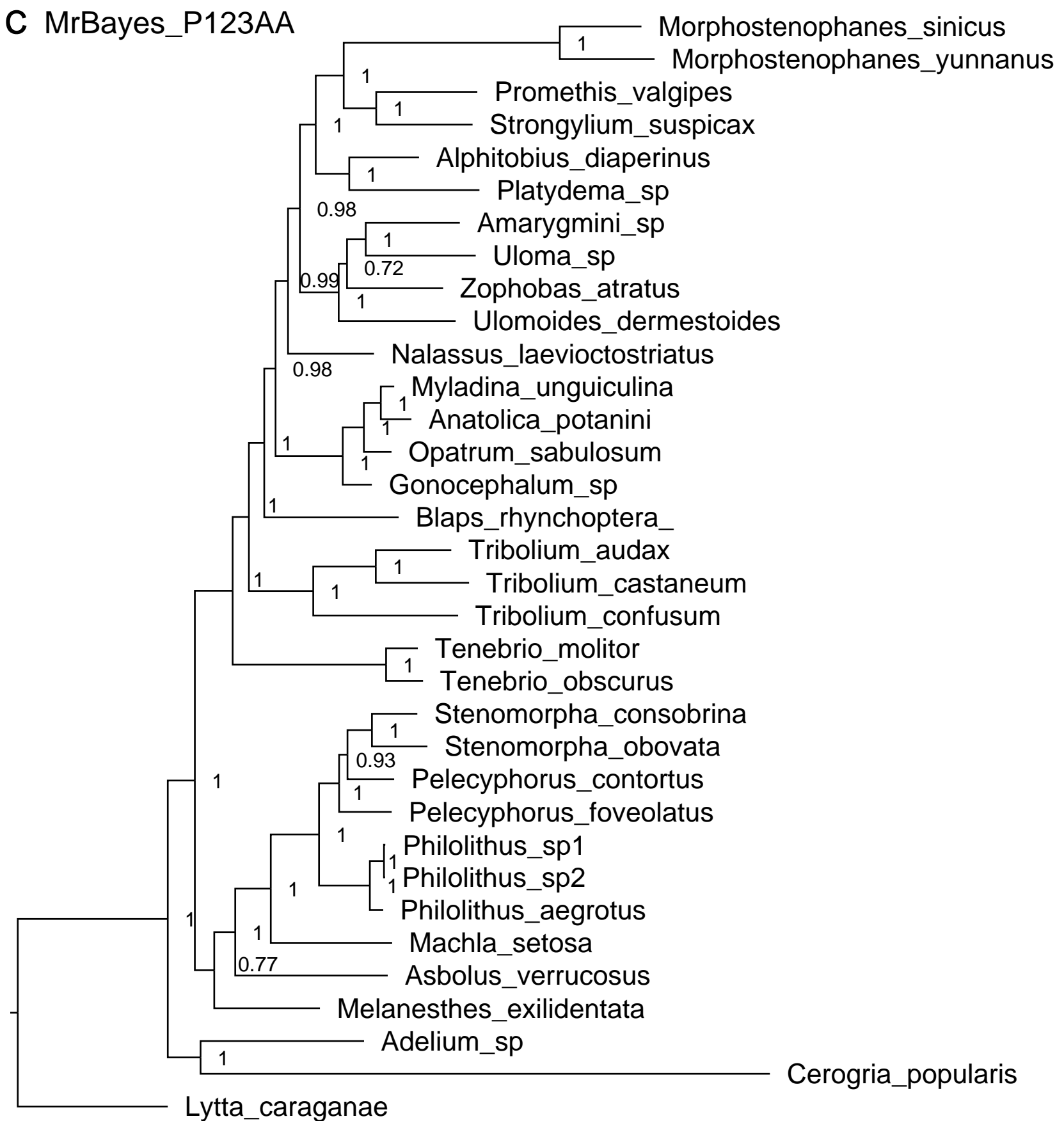

0.2

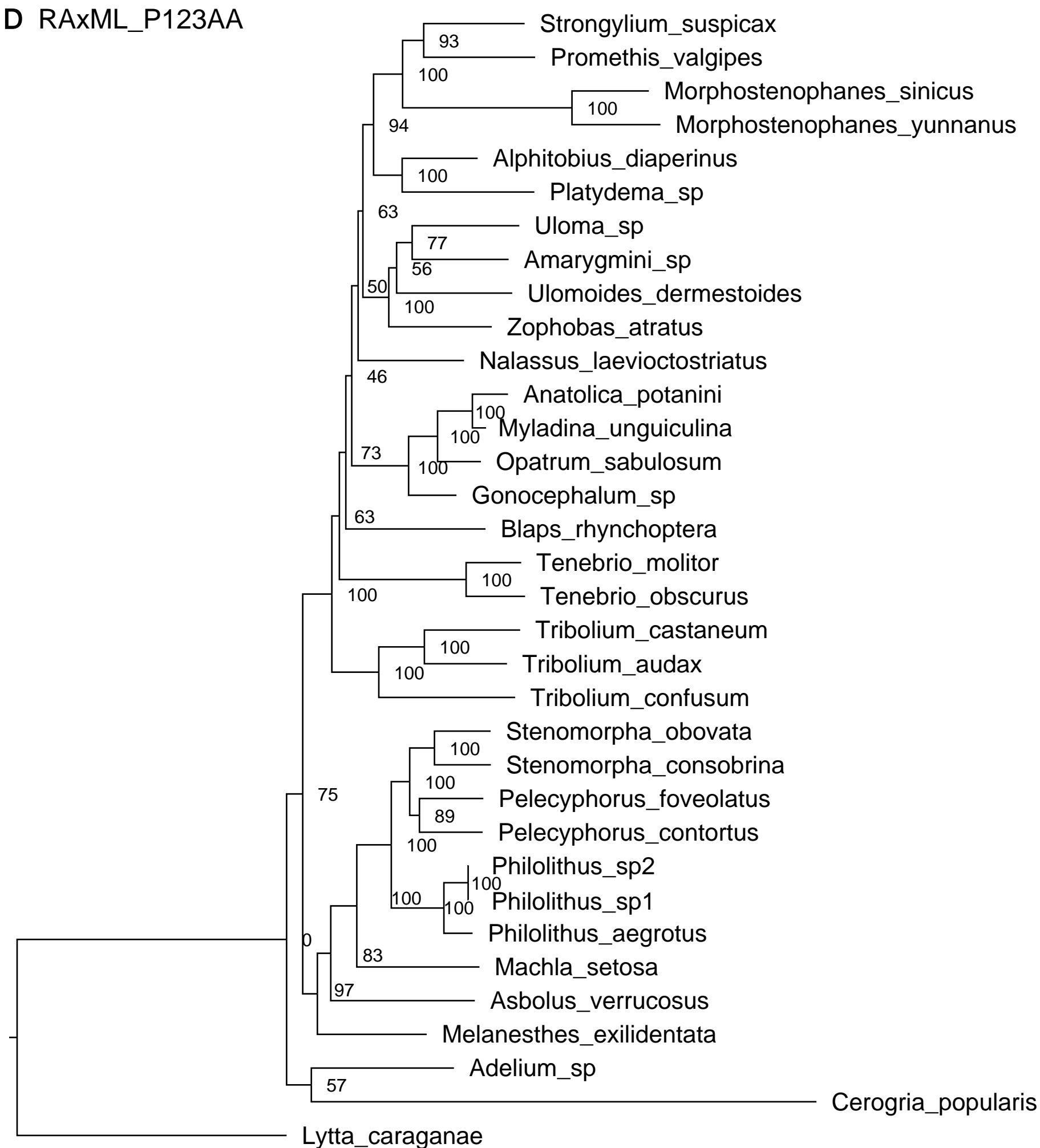

E MrBayes\_P123RNA

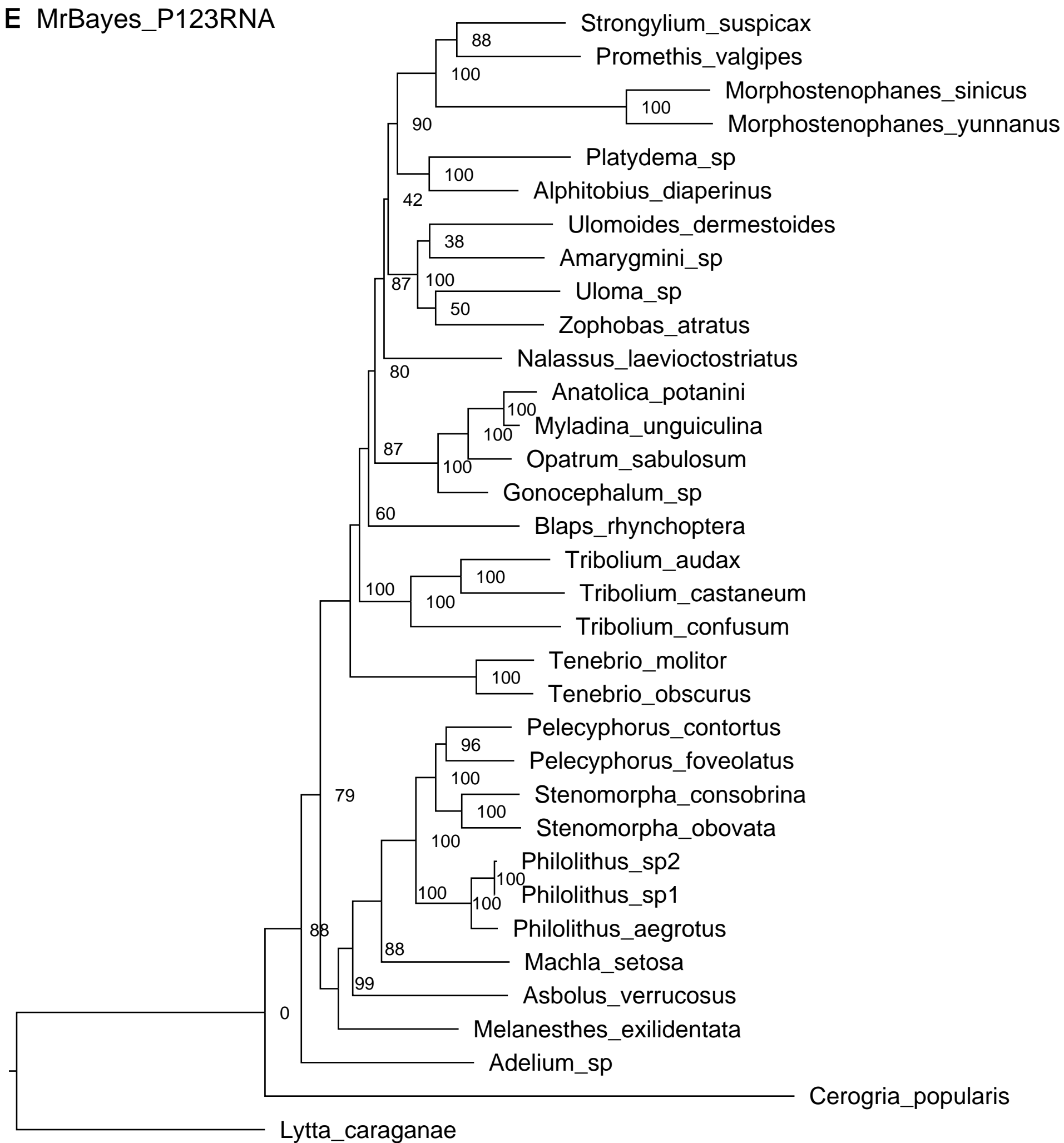

# F RAXML\_P123RNA

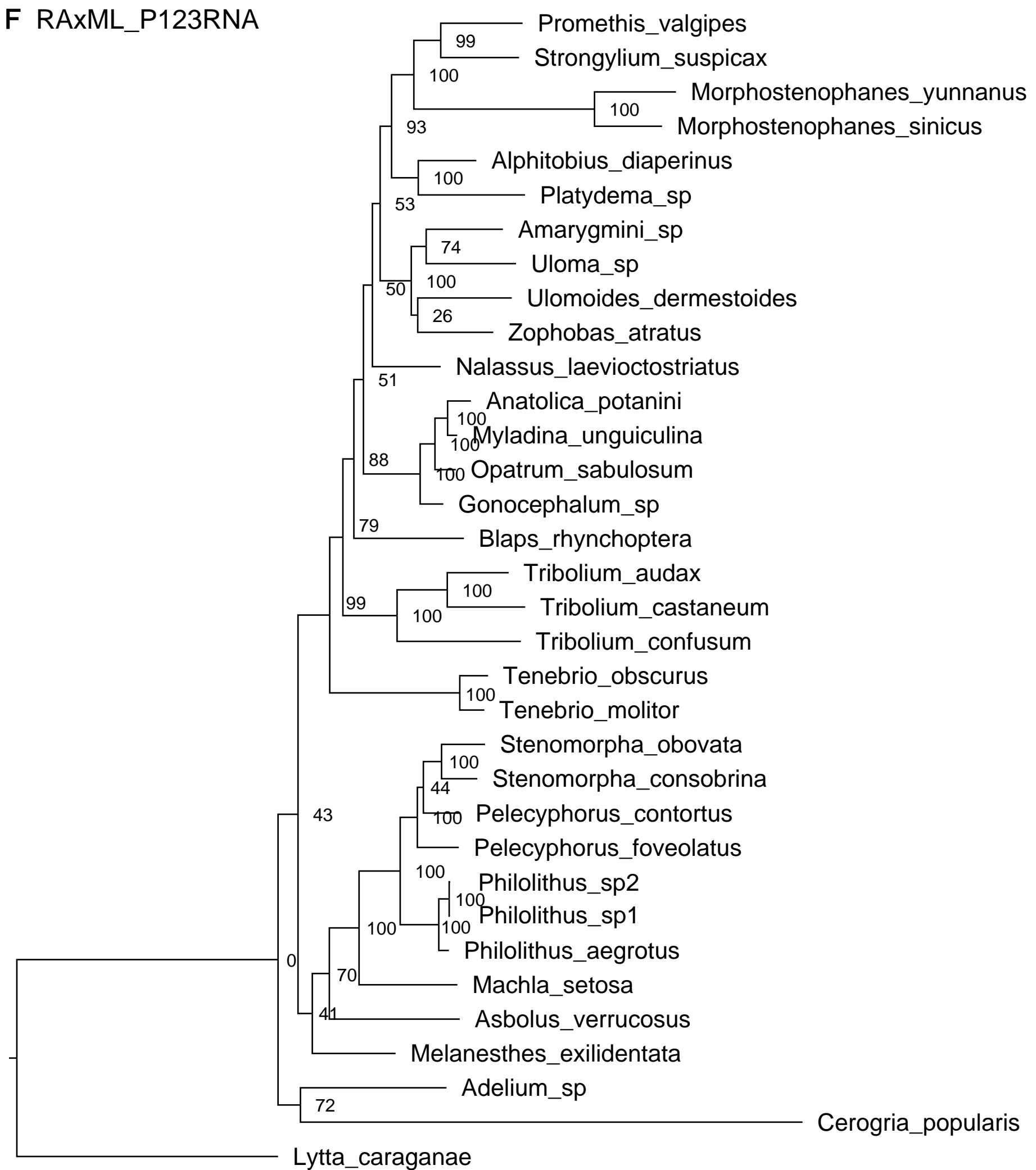

0.2

Supplement: Supplementary file 1 [file genes-14-01738-s001.zip › Figure S5 Phylogenetic results.pdf]
